# Undinarchaeota illuminate DPANN phylogeny and the impact of gene transfer on archaeal evolution

**DOI:** 10.1038/s41467-020-17408-w

**Published:** 2020-08-07

**Authors:** Nina Dombrowski, Tom A. Williams, Jiarui Sun, Benjamin J. Woodcroft, Jun-Hoe Lee, Bui Quang Minh, Christian Rinke, Anja Spang

**Affiliations:** 1 NIOZ, Royal Netherlands Institute for Sea Research, Department of Marine Microbiology and Biogeochemistry, and Utrecht University, P.O. Box 59, NL-1790 AB Den Burg, The Netherlands; 2 School of Biological Sciences, University of Bristol, Bristol, BS8 1TQ UK; 3 Australian Centre for Ecogenomics, School of Chemistry and Molecular Biosciences, The University of Queensland, Brisbane, QLD 4072 Australia; 4 Department of Cell- and Molecular Biology, Science for Life Laboratory, Uppsala University, SE-75123 Uppsala, Sweden; 5 Research School of Computer Science and Research School of Biology, Australian National University, Canberra, ACT 2601 Australia

**Keywords:** Computational biology and bioinformatics, Phylogenetics, Phylogenomics, Archaeal evolution

## Abstract

The recently discovered DPANN archaea are a potentially deep-branching, monophyletic radiation of organisms with small cells and genomes. However, the monophyly and early emergence of the various DPANN clades and their role in life’s evolution are debated. Here, we reconstructed and analysed genomes of an uncharacterized archaeal phylum (*Candidatus* Undinarchaeota), revealing that its members have small genomes and, while potentially being able to conserve energy through fermentation, likely depend on partner organisms for the acquisition of certain metabolites. Our phylogenomic analyses robustly place Undinarchaeota as an independent lineage between two highly supported ‘DPANN’ clans. Further, our analyses suggest that DPANN have exchanged core genes with their hosts, adding to the difficulty of placing DPANN in the tree of life. This pattern can be sufficiently dominant to allow identifying known symbiont-host clades based on routes of gene transfer. Together, our work provides insights into the origins and evolution of DPANN and their hosts.

## Abstract (web summary)

The evolutionary relationships within Archaea remain unresolved. Here, the authors used genomic approaches to study the Undinarchaeota, a previously uncharacterized clade of DPANN, shed light on their position in an updated archaeal phylogeny and illuminate the history of archaeal genome evolution.

## Introduction

Archaea represent one of the two primary domains of life^1–3^ and are thought to have played a major role in the evolution of life and origin of Eukaryotes^4–6^. While most archaea remain uncultivated, cultivation-independent approaches, such as single-cell and metagenomic sequencing, have revealed many previously unknown archaeal lineages in most environments on Earth and have changed our perception of archaeal functional and taxonomic diversity^7–10^. In particular, the Asgard^5^ and DPANN superphyla^11,12^ as well as a multitude of putative phylum-level lineages have been proposed in the archaeal domain over the last two decades but the phylogenetic relatedness and taxonomy of the various archaeal lineages remain a matter of debate^10^.

The DPANN radiation^12^, named after the first members of this group (Diapherotrites, Parvarchaeota, Aenigmarchaeota, Nanoarchaeota and Nanohaloarchaeota)^11^, comprises one of these recently proposed archaeal clades and is now thought to be comprised of at least ten (according to NCBI taxonomy) putative phylum-level lineages^13,14^. Most members of the DPANN archaea are characterized by small cell sizes and reduced genomes, which code for a limited set of metabolic proteins^14^. The few members that have been successfully enriched in co-culture were shown to represent obligate ectosymbionts dependent on archaeal hosts for growth and survival. For instance, members of Nanoarchaeota are ectosymbionts of various Crenarchaeota such as for example *Ignicoccus hospitalis*, *Sulfolobales* Acd1 and *Acidilobus* sp. 7A^15–20^, Micrarchaeota were found in co-culture with Thermoplasmates^21,22^ and Nanohaloarchaeota are dependent on halobacterial hosts^23^. Furthermore, evidence from FISH and co-occurrence analyses have suggested that Huberarchaeota may be ectosymbionts of members of the Altiarchaeota^24,25^. Yet, for most DPANN representatives, the identity of their potential symbiotic partners remains unclear.

Ever since the discovery of the first DPANN representative—*Nanoarchaeum equitans*, an ectosymbiont of *Ignicoccus hospitalis*^15^—the phylogenetic placement of putative DPANN clades in the archaeal tree have been uncertain^26^. While various phylogenetic analyses have indicated that DPANN may comprise a monophyletic radiation in the Archaea^9,11,27^, these have been debated^8,28,29^. In particular, analyses focusing on the placement of selected DPANN lineages in isolation, such as Nanoarchaeota and Parvarchaeota, relative to other Archaea, have led to the conclusion that these represent fast-evolving Euryarchaeota^28,29^. Furthermore, it is debated whether the free-living Altiarchaeota belong to the DPANN radiation, form an independent lineage or belong to Euryarchaeota^8,10,13,30,31^. A potential cause for these conflicting topologies is that DPANN are often found on long branches in phylogenetic trees; these long branches might result from compositional biases or fast evolutionary rates^32,33^ (as seen for obligate bacterial endosymbionts^34,35^) or might reflect genomic undersampling of the true diversity of this group^10^. These alternatives are difficult to distinguish because, in the absence of fossils or definitive geochemical traces in the fossil record, we lack a well-constrained timescale for archaeal evolution. Distantly related long-branching lineages can sometimes artificially group together on trees due to methodological artefacts, a phenomenon called long-branch attraction (LBA)^32^. Ways to ameliorate such artefacts include increased taxonomic sampling^36^, use of phylogenetic models less prone to LBA^37^, and the removal of fast-evolving or compositionally biased sites from the alignment^38^. Furthermore, it seems possible that horizontal gene transfers (HGT) between symbionts and hosts^39^ could impede correct phylogenetic inferences if not accounted for.

Several recent studies have revealed the presence of a thus far uncharacterized archaeal lineage referred to as the Uncultivated Archaeal Phylum 2 (UAP2)^14,40,41^, which seems to affiliate with DPANN archaea and thus may be key in resolving longstanding debates regarding archaeal phylogeny and the evolution of DPANN. In this study, we use a metagenomics approach to obtain additional genomes of members of the so far uncharacterized UAP2 and provide first insights into their metabolic repertoire and lifestyle. We implement comprehensive and careful phylogenomic techniques aimed at ameliorating phylogenetic artefacts that shed new light onto the evolutionary origin and phylogenetic placement of the various DPANN lineages, including UAP2, in an updated archaeal phylogeny. Furthermore, our work reveals major routes of horizontal gene transfer (HGT) across archaeal clades including among DPANN symbionts and their hosts.

## Results and discussion

### An uncharacterized archaeal phylum-level lineage in read archives

The generation of a large diversity of metagenome-assembled genomes (MAGs) representing archaeal and bacterial lineages across the tree of life has led to the definition of the tentative archaeal UAP2 phylum^41^. Considering our lack of insights into the biology of members of this lineage as well as its suggested key position in the archaeal tree, we aimed at obtaining a broader genomic representation of the UAP2. In particular, we screened publicly available metagenomes using ribosomal protein sequences of the previously reconstructed UAP2 MAGs and assembled and binned UAP2-positive samples yielding six additional MAGs belonging to the UAP2 lineage (Table 1, Supplementary Data 1 and 2, see Methods for details). Four of the newly assembled MAGs were recovered from metagenomes of a groundwater aquifer located adjacent to the Colorado River^42^, while the two others as well as six previously reconstructed MAGs, derived from metagenomes of marine waters in the Atlantic^41,43^ and Indian Oceans^44^ as well as the Mediterranean Sea^45^ (Supplementary Data 2). UAP2 representatives were detected in samples from various depths in the water column (85-5000 m), with fluctuating oxygen conditions (anoxic to oxic) and temperatures (sampling sites had temperatures from 18 up to 106 °C) (Supplementary Fig. 1, Supplementary Data 1). The MAGs, including previously published ones, are on average 78% complete (min: 55%, max: 91%) and show low signs of contamination (<5%) and strain heterogeneity (<2%). In total, they represent 2 high-quality (one from this study) and 10 medium-quality draft genomes according to genome reporting standards for MAGs assessed using a general archaeal marker protein set (Table 1, Supplementary Discussion)^46^. The UAP2 MAGs have small genomes with an average size of 0.66 Mbp, coding for an average of 750 proteins. They likely represent a distinct archaeal phylum-level lineage based on average amino-acid identity (AAI) comparisons with other archaeal taxa (Supplementary Fig. 2, Supplementary Data 3), phylogenetic analyses including a concatenated 16S-23S rRNA gene tree (Supplementary Figs. 3–5 and see below) as well as classification based on the Genome Taxonomy Database (GTDB) rank normalization (Table 1, Supplementary Data 2). Furthermore, the aquifer and marine UAP2 MAGs likely represent different orders according to GTDB-Tk^47^, which normalizes ranks using relative evolutionary divergence^48^. Based on two high-quality UAP2 MAGs (Table 1, Supplementary Data 2)^46^, we propose two type species; ‘*Candidatus* Undinarchaeum marinum’ (SRR4028224.bin17) and ‘*Candidatus* Naiadarchaeum limnaeum’ (SRR2090159.bin1129), representing the marine and aquifer UAP2 clade, respectively (see details below). Undines are water elementals described in the writings of the alchemist Paracelsus, while Naiads are nymphs residing in ponds, springs and other bodies of freshwater in Greek mythology.

**Table 1:** Genome characteristics of Undinarchaeota MAGs.

BinID	Order	Habitat	Length (Mbp)	GC (%)	Largest contig (bp)	No. of contigs	No. of proteins	16S	23S	5S	tRNA count	No. of markers	0	1	2	Completeness (%)	Contamination (%)	Heterogeneity (%)
SRR2090153.bin1042	Naiadarchaeales	Aquifer	0.72	37.7	12.286	144	809				15	149 (142)	61 (54)	87	1	65.0 (69.1)	0.93 (1.0)	0
SRR2090153.bin461	Naiadarchaeales	Aquifer	0.68	43.0	11.937	152	787				17	149 (142)	76 (69)	69	4	55.0 (58.6)	3.7 (3.92)	50
SRR2090159.bin1129^a^	Naiadarchaeales	Aquifer	0.98	37.9	97.397	52	1081		1	1	21	149 (142)	11 (4)	135	3	90.7 (96.0)	2.8 (2.9)	0
SRR2090159.bin1288	Naiadarchaeales	Aquifer	0.70	43.0	19.705	102	821				17	150 (142)	63 (56)	82	4	65.1 (69.3)	3.7 (3.9)	75
GCA_002502135.1	Undinarchaeales	Marine	0.73	39.5	74.625	23	819	1	1	1	19	151 (142)	23 (16)	126	0	79.9 (84.3)	0 (0)	0
GCA_002494525.1	Undinarchaeales	Marine	0.64	40.7	145.584	11	706		1	1	21	152 (142)	12 (5)	137	0	89.7 (95.1)	0 (0)	0
GCA_002495465.1	Undinarchaeales	Marine	0.61	41.4	75.044	19	696		1	1	19	153 (142)	21 (14)	128	0	82.6 (87.6)	0 (0)	0
GCA_002501985.1	Undinarchaeales	Marine	0.61	40.8	118.101	22	678			1	21	154 (142)	14 (7)	135	0	87.9 (93.1)	0 (0)	0
GCA_002687935.1	Undinarchaeales	Marine	0.67	41.6	173.317	11	752	1	1	1	21	155 (142)	12 (5)	135	2	89.7 (95.1)	1.9 (2.0)	100
SRR4028224.bin17^a^	Undinarchaeales	Marine	0.62	42.3	114.673	19	700	1	1	1	21	156 (142)	12 (5)	137	0	89.7 (95.1)	0 (0)	0
SRR5007147.bin71	Undinarchaeales	Marine	0.62	42.0	54.083	39	705			1	21	157 (142)	17 (10)	132	0	87.2 (92.5)	0 (0)	0
U_76725	Undinarchaeales	Marine	0.37	40.2	12.036	80	416				13	158 (142)	76 (69)	72	1	54.4 (58.1)	0.93 (1.0)	100

General genome statistics, including genome size, GC-content, contig number and other quality characteristics (presence of 16S, 23S and 5S rRNA genes). Additionally, the degree of genome completeness, contamination and strain heterogeneity was estimated using CheckM. 0-2: Number of single-copy marker proteins present in each genome. The CheckM results were investigated for markers commonly absent in DPANN archaea (Supplementary Discussion) and CheckM was rerun excluding seven marker proteins. The results of this analysis are shown in parentheses.

^a^Type species of Naiadarchaeales and Undinarchaeales.

### Description of new taxa

‘*Candidatus* Undinarchaeum’ (Un.din.ar.chae’um. N.L. n. *Undina* female water spirit or nymph (from L. fem. n. *unda* water, wave); N.L. neut. n. *archaeum* (from Gr. adj. *archaios* ancient) archaeon; N.L. neut. n. *Undinarchaeum* an archaeon of water origin).

*‘Candidatus* Undinarchaeum marinum’ (ma.ri’num. L. neut. adj. *marinum* of the sea, marine). Type material is the genome designated as SRR4028224.bin17 representing ‘*Candidatus* Undinarchaeum marinum’.

*Candidatus* Naiadarchaeum (Na.iad.ar.cha’eum. L. fem. n. *Naias*, -*adis* a water-nymph of springs and streams, Naiad from Greek mythology; N.L. neut. n. *archaeum* (from Gr. adj. *archaios* ancient) archaeon; N.L. neut. n. *Naiadarchaeum* an archaeon from the freshwater).

*‘Candidatus* Naiadarchaeum limnaeum’ (lim.nae’um. N. L. neut. adj. *limnaeum* (from Gr. adj. *limnaios* from the marsh, lake) living in the freshwater). Type material is the genome designated as SRR2090159.bin1129 representing ‘*Candidatus* Naiadarchaeum limnaeum’.

Based on these genera, we further propose the families ‘*Candidatus* Undinarchaeaceae’ fam. nov. and ‘*Candidatus* Naiadarchaeaceae’ fam. nov., the orders ‘*Candidatus* Undinarchaeales’ ord. nov. and ‘*Candidatus* Naiadarchaeales’ ord. nov., the class ‘*Candidatus* Undinarchaeia’ class nov., and the phylum ‘*Candidatus* Undinarchaeota’ phylum nov. (see Supplementary Discussion for additional details).

### Undinarchaeota branch between two major DPANN clades

Initial phylogenetic analyses placed Undinarchaeota (formerly UAP2) as a sister lineage to all other DPANN archaea in unrooted trees^13,14,41^. If correct, this placement could give important insights into the timing of DPANN evolution and the nature of the putative last DPANN common ancestor. However, this deep-branching position was poorly supported^14,41^. In order to resolve the phylogenetic relationship of Undinarchaeota and DPANN archaea as well as to test the monophyly of the DPANN radiation, we performed in-depth phylogenetic analyses (Supplementary Discussion, Methods).

We began by updating the taxon sampling in three marker protein datasets including those used by PhyloSift and GTDB^9,47,49^ and inferred single-protein trees for each marker to evaluate phylogenetic congruence and detect contaminant sequences and HGTs (Methods, Supplementary Data 4 and 5). Since an initial manual inspection revealed extensive incongruence among markers, we developed a marker protein ranking scheme to compare proteins and datasets systematically and without a priori assumptions regarding archaeal phylogeny above the rank of order-, class- or phylum (details in Methods, Supplementary Discussion). In brief, we first removed markers, which violated archaeal monophyly (Methods, Supplementary Data 4 and 5). Subsequently, we ranked marker genes according to the extent to which they supported the monophyly of well-established archaeal phylum-, class and order-level lineages but not the relationship of these lineages to each other (Supplementary Data 4 and 5) (Methods, Supplementary Discussion)^5,47,49^. Thus, since DPANN monophyly remains actively debated^8,10–12,26–29^, we neither penalize marker genes for failing to recover the monophyly of the superphylum as a whole, nor the placement of certain DPANN lineages with other DPANN lineages or archaeal taxa.

While top-ranked markers had low numbers of so-called splits, i.e. recovered accepted archaeal lineages as monophyletic clades, low ranking markers were highly incongruent with individual members of accepted lineages not grouping together (see Methods for details, Supplementary Data 4 and 5). Raw and aligned sequences of top-ranked markers were longer (p-values: 2.088e-05 and 1.224e-07, respectively) (Supplementary Fig. 6a, b) than those of low-ranked markers and had higher overall bootstrap supports (*p*-value: 1.35e-12) (Supplementary Fig. 6c) suggesting that lack of phylogenetic signal in low-ranked markers may contribute, at least in part, to their failure to recover established archaeal lineages. However, low-ranked markers showed moderate to strong support for among others the placement of specific DPANN with their host lineages (e.g. Nanoarchaeum with Crenarchaeota, Nanohaloarchaeota with Halobacteria) (Supplementary Data 4 and 5, Supplementary Fig. 7) indicating that a phylogenetic signal does contribute to topological incongruencies. The support for these relationships varied with different low-ranked markers providing support for different DPANN-host relationships (Supplementary Fig. 7, Supplementary Data 4). Altogether, this suggests that several universal archaeal genes, including those coding for ribosomal proteins and other core elements of the genetic machinery, may have undergone interlineage gene transfers during archaeal diversification.

**Fig. 1:**
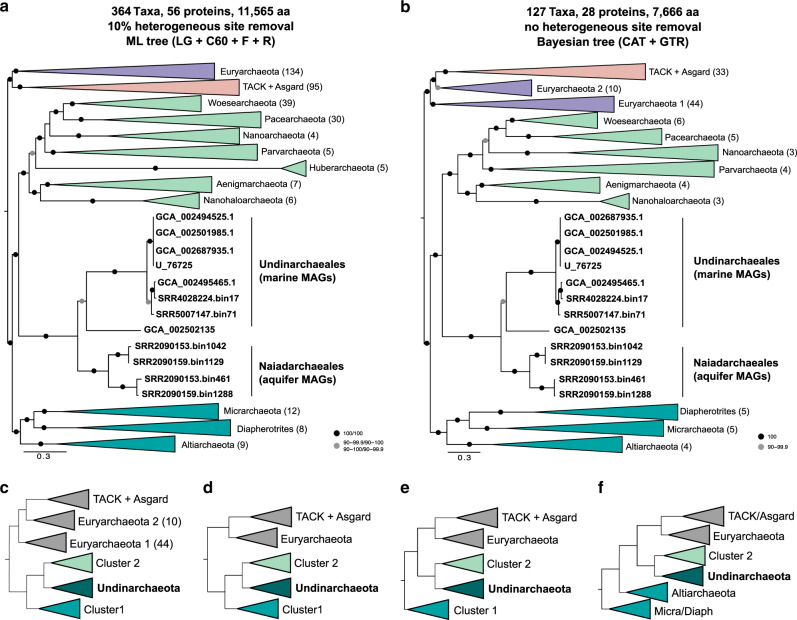
Phylogenetic placement of Undinarchaeota. **a** Maximum-likelihood phylogenetic analysis (LG + C60 + F + R model) of the concatenated 50% top-ranked marker proteins (*n* = 56) and 364 taxa set. For this alignment 10% of the most heterogeneous sites were removed using chi2 pruning. The full tree is shown in Supplementary Fig. 19. **b** Bayesian phylogenetic tree (CAT + GTR model) of an alignment generated with the 25% top-ranked marker proteins (*n* = 28) and 127 taxa set. The full tree is shown in Supplementary Fig. 10. Euryarchaeota 1 includes all Euryarchaeota with the exception of Theionarchaea, Thermococci, Persephonarchaea and Hadesarchaea, which are clustered in Euryarchaeota 2. Scale bar: Average number of substitutions per site. **c**–**f** Possible positions of the archaeal root inferred using bacteria as an outgroup (**c**) or using the nonreversible model in IQ-TREE 2 **d**–**f**. All of these methods recover a clade of Undinarchaeota and Cluster 2 DPANN, consistent with synapomorphies including a fused DNA primase and a reduced exosome that lacks Csl4.

We now have increased power to detect such transfers due to the expanded taxonomic sampling of the archaeal domain compared to previous analyses. However, the low information content contained in single-protein alignments as well as low average bootstrap support in corresponding trees (see Methods, Supplementary Fig. 6c, Supplementary Data 4 and 5), motivates the use of protein concatenations for phylogenetic reconstructions. A large number of gene transfers among markers can mislead phylogenomic analyses because current concatenation and supertree methods assume that all genes evolve on the same underlying tree. To ameliorate the impact of incongruent markers on our inferences, concatenated phylogenies were inferred from the 25 and 50% top-ranked marker proteins, which correspond to those markers with lowest numbers of splits and in turn potential HGTs. As compositionally heterogeneity across sites is a pervasive feature of archaeal sequence evolution^9,11,27^, we used site-heterogeneous mixture models in our focal analyses in both maximum likelihood (IQ-TREE^50^) and Bayesian (PhyloBayes^51^) frameworks, in combination with alignment recoding and filtering of compositionally biased and fast-evolving sites (Supplementary Data 6, Supplementary Figs. 8–58, Methods, Supplementary Discussion). Our analyses consistently recovered the clanhood^52^ of the DPANN archaea (including Undinarchaeota) as a whole; i.e. all DPANN archaea clustered together (formed a clan) on the unrooted tree (Fig. 1a, b). Furthermore, our inferences based on curated marker set alignments consistently suggested that Undinarchaeota form a distinct lineage that branches between two other DPANN clans (sequence clusters on the unrooted tree^52^) with maximum statistical support (Fig. 1a, b, Supplementary Figs. 8–47, Supplementary Data 6; Supplementary Discussion). These clans comprised the Altiarchaeota, Micrarchaeota and Diapherotrites (hereafter referred to as DPANN Cluster 1) and all remaining members of the DPANN (Woesearchaeota, Pacearchaeota, Parvarchaeota, UAP1, Nanoarchaeota, Huberarchaeota, Aenigmarchaeota and Nanohaloarchaeota) (hereafter referred to as DPANN Cluster 2), respectively (Fig. 1a, b). Finally, in all phylogenies, Undinarchaeota formed two GTDB-level orders, consisting of aquifer-derived and ocean-derived MAGs, i.e. the Naiadarchaeales and Undinarchaeales, respectively (Fig. 1a, b; Supplementary Data 2).

Next, we compared these results with phylogenetic inferences based on the 25 and 50% most incongruent markers (Supplementary Data 4 and 5), which were inferred to have experienced high rates of interlineage transfers or were otherwise affected by conflicting phylogenetic signals (Supplementary Figs. 48–51). In agreement with our predictions, these analyses yielded phylogenetic trees with various highly supported relationships among unrelated taxa (Supplementary Discussion). For instance, analyses based on the 25% lowest ranking markers recovered Nanoarchaeota as members of the TACK^53^ archaea (Supplementary Fig. 49, 127 taxa set) and Nanohaloarchaeota as a sister lineage of Halobacteria either as a separate cluster (Supplementary Fig. 48, 364 taxa set) or with DPANN archaea (Supplementary Fig. 49, 127 taxa set), in agreement with known symbiont-host relationships^16,17,23^. This is particularly notable because we did not a priori penalize trees in which certain DPANN lineages branch with certain other archaeal lineages (Supplementary Data 4 and 5). In turn, these analyses suggest that conflicting results regarding the placement of certain DPANN lineages, may, at least in part, be due to inadequate taxon sampling and the use of a large number of markers affected by host-symbiont HGT. For instance, Nanohaloarchaeota may artificially be drawn towards the Euryarchaeota^28^ when marker sets include too many proteins that were affected by symbiont-host transfers.

Compositional biases in protein sequences can also lead to artefacts in phylogenetic reconstructions^54,55^. To assess the reliability of the inferred placement of Undinarchaeota based on our top-ranked marker protein sets and to ameliorate remaining biases, we subjected the curated alignment to different data treatments including removal of compositionally heterogeneous and fast-evolving sites (see Methods for details, Supplementary Data 6, Supplementary Figs. 15–24 and 32–42). Removal of compositionally biased sites resulted in notable changes in the tree topology. In particular, the originally inferred sister relationship between Halobacteria and Methanonatronarchaeia^56^ was supported only in analyses based on the original nontreated alignment; removal of 10% or more of the most biased sites instead supported a placement of Methanonatronarchaeia basal to Archaeoglobales, Methanomicrobia and Halobacteria (Supplementary Figs. 15 and 19), in agreement with more recent work^57^. However, the placement of Undinarchaeota relative to the DPANN Cluster 1 and Cluster 2, as well as the monophyly of each of these clusters, remained stable irrespective of the fraction of heterogeneous (10–40% of sites) or fast-evolving (10–40% of sites) sites removed, suggesting that our inferences are not an artefact of compositional or per-site substitution rate biases.

Finally, we reconstructed phylogenies using a recently-developed nonreversible substitution model that captures asymmetries in the exchange rates between amino acids^58^ to investigate the position of Undinarchaeota relative to the root of the archaeal tree (Fig. 1c, Supplementary Figs. 12,13,21,24,26–27,38 and 41, Supplementary Data 6). This method does not rely on an outgroup and therefore avoids potential LBA artefacts associated with the use of distantly related bacterial sequences to root the archaeal tree^27^. Notably, all our analyses recovered a monophyletic clade of Asgard, TACK and Euryarchaeota with the root being excluded from within this clade with high statistical support (100%). However, the nonreversible model failed to strongly resolve the root position within the DPANN radiation. In particular, the maximum-likelihood root was placed either (a) between all DPANN (including Cluster 1 and 2 as well as Undinarchaeota) on one side and all other Archaea on the other side (Fig. 1c, d, Supplementary Data 6, Supplementary Figs. 12–13), (b) between the Cluster 1 and all other archaea (Fig. 1e, Supplementary Data 6, Supplementary Figures 26, 27 and 38) or (c) between a cluster comprising Micrarchaeota-Diapherotrites and the rest of the Archaea (Fig. 1 f, Supplementary Table 6, Supplementary Figs. 21, 24 and 41). However, none of these root positions inferred using nonreversible models received high bootstrap support. Rooting using a bacterial outgroup recovered a root between a monophyletic DPANN clade and the rest of the Archaea with moderate to high bootstrap support (94% ultrafast bootstrap^59^, 98% SH-like aLRT support^60^; Supplementary Fig. 58), consistent with previous results^27^. Thus, our analyses provide strong support for the clanhood of DPANN archaea including Undinarchaeota, but do not confidently resolve the position of the archaeal root either within that clan, or between DPANN and other Archaea^9,13,27^.

### Synapomorphies of Undinarchaeota and Cluster 2 DPANN

To further assess the phylogenetic placement of the Undinarchaeota lineage, we surveyed the genomes of DPANN lineages for gene content synapomorphies (shared derived characters) that might enable us to distinguish competing hypotheses for the archaeal root. Similar to other DPANN lineages, Undinarchaeota MAGs encode most proteins involved in replication, transcription, translation and repair (Supplementary Data 7–10, Supplementary Figs. 59–63, Supplementary Discussion). While Undinarchaeota did not share specific features with any of the other archaeal clades, we identified candidate synapomorphies supporting a monophyletic clade comprising Undinarchaeota and Cluster 2 DPANN. Specifically, members of these lineages lack genes encoding the exosome component Csl4, which is present in Cluster 1 DPANN and most other archaea (Supplementary Fig. 63). The archaeal exosome is thought to consist of four subunits: Rrp41 and Rrp42 form the core ring structure, and Csl4 and Rrp4 constitute the rRNA-binding cap^61^. In spite of the absence of Csl4, Undinarchaeota and Cluster 2 DPANN archaea encode all other subunits of the complex (Rrp4/41/42) suggesting a structural or functional difference of their exosome.

Furthermore, Undinarchaeota and Cluster 2 DPANN share a synapomorphy related to the archaeal DNA primase^62^. Previous work^63^ has suggested that, while DNA primases of most Archaea (including those of the Micrarchaeota) are composed of two subunits encoded by *pri*S and *pri*L, some DPANN lineages (at that time the Nanoarchaeota, Nanohaloarchaeota and Parvarchaeota), were found to possess a *pri*S-*pri*L fusion gene. Our analyses, which includes a larger genomic representation of DPANN archaea, revealed that representatives of the DPANN Cluster 1 consistently encode canonical *pri*S and *pri*L genes, while all Undinarchaeota and DPANN Cluster 2 archaea have a fused version (Supplementary Data 11). Note that *pri*S and *pri*L arose from an ancestral duplication and are thus homologous. A phylogenetic analysis of all PriS and PriL subunits (after splitting the fused version), revealed that the fusion likely occurred at the origin of the Undinarchaeota and DPANN Cluster 2 (100/100 and 91.3/99 bootstrap support for PriS and PriL, respectively; Supplementary Fig. 59).

Consistent with our phylogenetic analyses, these findings support a clade containing Undinarchaeota and DPANN Cluster 2 as sister lineages from which the archaeal root is excluded. It will be interesting to experimentally investigate the functional implication of the identified synapomorphies (exosome component loss, DNA primase fusion) and determine whether they could have played a role in reductive genome evolution in Undinarchaeota and DPANN Cluster 2 archaea.

### Putative fermentative lifestyle and auxotrophies

**Fig. 2:**
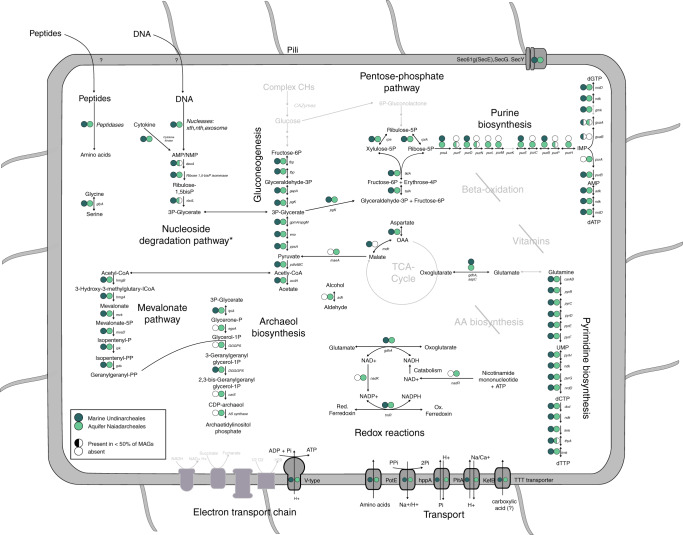
Metabolic characteristics of Undinarchaeota projected on key pathways. Full circles: Gene of interest is present in all or more than 50% of genomes. Half circles: Gene of interest is present in less than 50% but found in at least one Undinarchaeota MAG. Open circle: Gene is absent in all Undinarchaeota MAGs. Dark green: MAGs belonging to the marine Undinarchaeales. Light green: MAGs belonging to the aquifer Naiadarchaeales. Grey: Missing genes/pathways. *deoA was only present in one Naiadarchaeales MAG and genes encoding RuBisCO were only found in two out of four Naiadarchaeales MAGs and it remains to be determined whether this is due to genome incompleteness or a sign of genome streamlining. A detailed list of genes encoded by Undinarchaeota can be found in Supplementary Data 7–9.

Catabolism: Comparative genome analyses and inference of the metabolic potential of Undinarchaeota (Supplementary Data 7, 8 and 12–15, Supplementary Discussion for details), suggest that representatives of this clade likely rely on fermentative processes for energy conservation (Fig. 2). In particular, the presence of the lower Embden–Meyerhof and non-oxidative pentose-phosphate pathway but absence of most genes coding for enzymes of the tricarboxylic acid (TCA) cycle suggest that Undinarchaeota could generate ATP through fermentation of pyruvate to acetate. Simple carbohydrates, such as pyruvate, could perhaps be taken up by passive diffusion^64^. Furthermore, some members of the Naiad- and Undinarchaeales may be able to use extracellular DNA as growth substrate (Fig. 2). For example, most representatives of the Undinarchaeota encode the complete nucleoside degradation pathway^65–67^(Supplementary Discussion), including an AMP phosphorylase (DeoA), ribose 1,5-bisphosphate isomerase and Group-III ribulose 1,5-bisphosphate carboxylase (RbcL; RuBisCO) (Supplementary Fig. 61). In fact, many DPANN representatives have been reported to harbor a RuBisCO homolog and certain members have been suggested to be able to use nucleosides as substrates^67,68^. Undinarchaeota may import DNA via pili encoded by all Undinarchaeota MAGs and subsequently degrade those using their encoded nucleases (Supplementary Discussion)^69,70^. Intermediates of the nucleoside degradation pathway, such as glycerate-3-phosphate, may subsequently be channeled into the lower glycolytic pathway and contribute to energy conservation by an ATP synthesizing acetate-CoA ligase (acdA). Other products (e.g. glyceraldehyde-3P and fructose-6P produced via gluconeogenesis) may be further metabolized through the non-oxidative pentose-phosphate pathway allowing the synthesis of cellular building blocks such as pyrimidines and purines. It is however notable that group-III-like RuBisCO homologs encoded by Undinarchaeales MAGs have mutations in two positions of the RuBisCO motif^65–68^. In turn, it remains to be determined whether RuBisCO has retained its canonical function in these members of the Undinarchaeales and indeed enables growth on nucleosides (Supplementary Fig. 61; Supplementary Discussion). Considering the limited set of predicted proteins involved in central carbon metabolism, experimental verification will be needed to assess whether the encoded pathways provide sufficient ATP to sustain the energy metabolism of the various Undinarchaeota representatives.

**Fig. 3:**
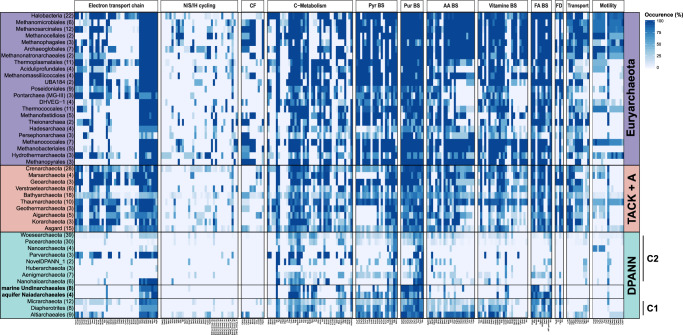
Occurrence of key metabolic proteins across major archaeal lineages. Protein occurrence was calculated by detecting key proteins of interest across 364 archaeal genomes and calculating the occurrence in percent across the total number of genomes included in each phylogenetic cluster based on a presence/absence table. Number in parentheses: number of genomes included in each individual phylogenetic cluster. CF Carbon fixation. FD Fatty acid degradation. C1/C2 DPANN Cluster 1/2. A table listing gene occurrences in archaeal lineages can be found in Supplementary Data 9 and a list of metabolic genes shown in this plot can be found in Supplementary Data 24.

Anabolism: Even though all representatives of the Undinarchaeota encode a near complete gluconeogenesis pathway (Fig. 2) including the potentially ancient bifunctional fructose 1,6-bisphosphate (FBP) aldolase/phosphatase, which would allow the synthesis of fructose-6-phosphate^71^, many other biosynthesis pathways are incomplete. For instance, while the Naiadarchaeales MAGs encode all proteins required to synthesize archaeal-type ether lipids, lipid biosynthesis pathways are incomplete in Undinarchaeales MAGs, which lack key genes for the conversion of glycerone-phosphate to archaetidylinosytol-phosphate, in spite of the presence of the archaeal mevalonate pathway^9^ in representatives of both lineages. Incomplete pathways for lipid biosynthesis are particularly common in DPANN Cluster 2 representatives (incl. *N. equitans*) (Fig. 3, Supplementary Fig. 64)^9,13,14,72,73^ and the characterization of the *N. equitans* - *I. hospitalis* symbiotic system has confirmed the exchange of lipids between symbiont and host^74^. Thus, while members of the Naiadarchaeales may synthesize their own lipids, Undinarchaeales representatives may depend on an external source of archaeal or bacterial lipids or intermediates in spite of the presence of the mevalonate pathway and their ability to synthesize geranylgeranyl diphosphate (Supplementary Discussion). Similarly, the lack of several genes coding for enzymes of the purine biosynthesis pathway in members of the Undinarchaeales but not Naiadarchaeales, indicates that the former are also dependent on an external source of inosine monophosphate (IMP) or other intermediates of the purine biosynthesis pathway (Figs. 2–3, Supplementary Data 7–9, Supplementary Discussion).

Furthermore, all Undinarchaeota representatives lack genes encoding proteins involved in amino acid and vitamin biosynthesis. Notably, and in agreement with several other potentially symbiotic DPANN archaea^13,14^, all Undinarchaeota representatives seem to contain a limited set of genes for transporters such as amino-acid transporter PotE and uncharacterized di/tri-carboxylate transporters (Supplementary Discussion, Figs. 2–3), suggesting that they are unable to acquire all essential building blocks directly from the environment. In turn, members of the Undinarchaeota seem to depend on partner organisms to provide compounds that cannot be synthesized or taken up from the environment using transporters. Key differences among the biosynthetic capabilities of members of the Naiad- and Undinarchaeales, may translate into varying substrate requirements and demands from potential host organisms.

Cell–cell interactions: Consistent with a host-dependent lifestyle, we detected several proteins with domains known to be involved in cell–cell interactions that are common among symbionts^14^ (Supplementary Discussion). While Undinarchaeota lack genes for ankyrin domain proteins and only encode a small number of beta propeller/WD40 domain proteins, the proteome of members of the Naiadarchaeales comprises diverse proteins with immunoglobulin domains, while Undinarchaeales encode Concanavalins/LamG domain proteins (arCOG07813) (Supplementary Data 16 and 17). Homology modeling and structure predictions suggested that these proteins might encode potential cell adhesion proteins (Supplementary Data 18 and 19) and in turn may be involved in attachment or biofilm formation in Undinarchaeota. Notably, the complete absence of LamG domain proteins in the Naiadarchaeales representatives indicates that members of the two Undinarchaeota orders might rely on different mechanisms mediating potential symbiont-host interactions.

**Fig. 4:**
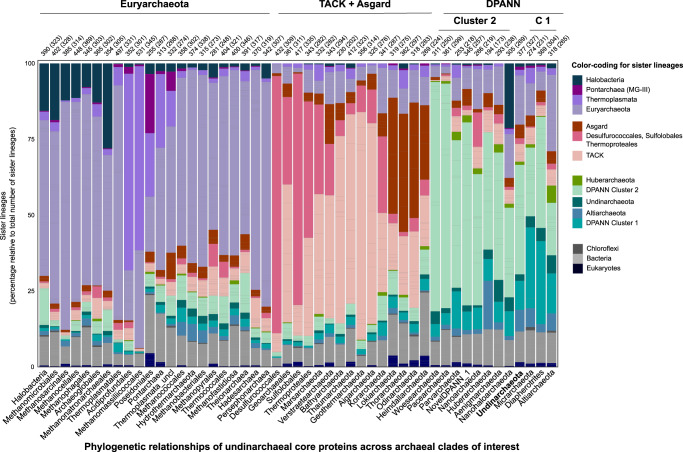
Sister-group relationships in phylogenies of core undinarchaeal proteins reveal host-symbiont gene sharing. The plot is based on phylogenetic analyses of 520 core Undinarchaeal proteins (shared across at least three MAGs of the Undinarchaeota), which included a representative set of archaeal, bacterial and eukaryotic genomes (*n* = 3482 taxa). It plots, for each archaeal clade (*x*-axis), the relative frequency with which other groups are recovered as the closest sister lineage, averaged over 520 protein trees and 1000 bootstrap replicates. (see Supplementary Data 20 for a list of all major archaeal clades of interest). The total number of sister lineages observed for a given clade is indicated on top of each bar graph together with the number of trees in which given clade occurred. Supplementary Data 20 and 21 as well as our data repository provide accompanying data for the HGT analysis for Undinarchaeota and other archaea as well as custom scripts.

Gene repertoires and reductive genome evolution in DPANN: The presence and absence patterns of genes involved in core metabolic pathways of Undinarchaeota MAGs show similar trends as seen in DPANN Cluster 2 archaea further supporting the sisterhood of this clade (Fig. 4, Supplementary Data 7–9, 12). For instance, most DPANN Cluster 2 archaea lack genes involved in core metabolic pathways, such as the electron transport chain, carbon fixation other than the RuBisCO gene, as well as transport and motility genes (Fig. 3)^9,13–16,23,25,39,75^. While Undinarchaeota seem to have more complete pathways than many of the DPANN Cluster 2 representatives, they appear metabolically less flexible than several members of DPANN Cluster 1 (Fig. 3)^30,31,76,77^. For instance, members of the DPANN Cluster 1 have more complete nucleoside and lipid biosynthesis pathways and include free-living organisms. In particular, representatives of the Altiarchaeota have been suggested to comprise autotrophic archaea that may use the Wood–Ljungdahl pathway for carbon fixation^24,25,30^ and while this lineage includes symbionts, these do not seem to be obligate^78,79^. In fact, Altiarchaeota have recently been found to include members that likely serve as hosts for Huberarchaeota belonging to DPANN Cluster 2^25^. Furthermore, at least some members of the Diapherotrites have been suggested to be capable of a fermentative free-living lifestyle^77^. However, in spite of overall gene repertoire patterns being consistent with results from our phylogenetic analyses, there is a large variation in gene content and extent of genome reduction within DPANN lineages^14^. Thus, our analyses further support the notion that while reductive genome evolution may have characterized the evolution of Undinarchaeota and DPANN Cluster 2 archaea already at the time of their divergence, the extent of streamlining varies widely and seems to have occurred in parallel in different lineages.

### Insights into putative interaction partners of Undinarchaeota

Genomic analyses of the first members of the Nanoarchaeota^39^ as well as our marker protein analyses have indicated that DPANN symbionts may have exchanged genes with their hosts. Furthermore, co-occurrence patterns have recently allowed to pinpoint Altiarchaeota as host for the Huberarchaeota^24,25^. Thus, to shed light onto potential interaction partners of the Naiad- and Undinarchaeales, respectively, we have inferred routes of horizontal gene transfer and generated proportionality networks.

In particular, we reconstructed phylogenies of proteins present in at least three Undinarchaeota genomes (520 genes total) and analyzed sisterhood relationships among taxonomically distinct lineages including a reference set of 364 archaeal, 3020 bacterial and 100 eukaryotic genomes (Fig. 4, Supplementary Data 20–22, Methods, Supplementary Discussion). Using this approach, we were able to successfully detect signals of HGT among known host-symbiont pairs. For instance, our analysis revealed several instances of sisterhood relationships of Nanoarchaeota and Huberarchaeota clades, with their respective hosts belonging to the Crenarchaeota and Altiarchaeota, respectively (i.e. 11% and 16% of all sisterhood relationships for these two clades above a normalized occurrence of >0.3, respectively). Furthermore, our phylogenies suggest that in several instances Nanohaloarchaeota and Halobacteria formed sister-groups suggesting that these lineages have exchanged proteins horizontally (i.e. 21% and 11% of all sisterhood relationships for these two clades, respectively) (Fig. 4), which is in agreement with our marker gene analyses (Supplementary Data 4 and 5). However, some of the sisterhood relationships could also be due to compositional biases as a result of protein sequence adaptation to high salinity environments or phylogenetic artefacts. Notably, Undinarchaeota does not show a dominant fraction of genes shared with a specific lineage, i.e. most proteins seem to cluster with other DPANN lineages (Fig. 4) or in some cases with potentially deeply branching Hydrothermarchaeota or group 1 methanogens (Supplementary Data 4, 21, 22). The few Undinarchaeota genes that may have been shared with distantly related taxa encoded proteins that grouped with Bathyarchaeota and Thermoplasmata (i.e. including Poseidoniales (MG-II) and Pontarchaea (MG-III) (Supplementary Discussion, Supplementary Data 4 and 21). For instance, the manual inspection of respective trees revealed that certain Undinarchaeota, in particular marine Undinarchaeales, may have exchanged core genes with members of the Pontarchaea, including a gene encoding ribosomal protein S19 (arCOG04099, TIGR01025) (Supplementary Data 4; Supplementary Fig. 65a, b). Yet, due to the small fraction of putative HGTs with any of these non-DPANN lineages, it remains to be assessed whether these patterns are genuine or result from phylogenetic artefacts, lack of partner organisms in current databases or Undinarchaeota forming less intimate interactions than DPANN Cluster 2 archaea.

Finally, we used a read-based co-proportionality analysis to assess whether the abundance of Undinarchaeota MAGs co-varied with other archaeal and bacterial genomes^80^ (see Methods for details). Due to the low number of available samples for our inferences of co-proportionality, this analysis did not detect any significant co-proportionality patterns for members of the Naiadarchaeales. However, it did indicate that marine Undinarchaeales may co-vary with three genomes of the Chloroflexi, all belonging to the order Dehalococcoidales (Supplementary Fig. 66, Supplementary Discussion). Considering that we could identify only few HGTs between members of these groups, it remains unclear, whether certain members of the Undinarchaeota and Chloroflexi have intricate interactions (Supplementary Fig. 65c, Supplementary Discussion).

Altogether, in light of these results and since Naiad- and Undinarchaeales differ with respect to putative proteins involved in cell–cell interactions (Supplementary Data 16–19) and certain metabolic features (e.g. the presence versus absence of a complete lipid biosynthesis pathway, (Fig. 2, Supplementary Discussion), it seems likely that members of these orders interact with different microbial partners. Further analyses, such as fluorescence in situ hybridization, can help to identify the organisms engaging in symbiotic interactions with Undinarchaeota. For instance, it will be important to determine their host-specificity and test whether certain members of marine Undinarchaeales indeed interact with members of the Chloroflexi or Pontarchaea. Together with previous findings^81^, our results suggest that certain DPANN symbionts could have non-archaeal partners highlighting the importance of taking into account both bacterial and archaeal community compositions when inferring potential co-occurrences between DPANN and other organismal groups.

### Conclusions

We provided an updated view on the evolution of DPANN archaea by investigating the phylogenetic placement and metabolic potential of the Undinarchaeota, a previously uncharacterized archaeal lineage whose members have small genomes and limited metabolic gene repertoires indicative of various auxotrophies. Its members, classified into the two order-level lineages Undinarchaeales and Naiadarchaeales, may lead a symbiotic lifestyle and depend on potentially different partner organisms for growth and survival similar to other members of the various DPANN representatives^9,13,14^. In addition to insights into the biology of members of the Undinarchaeota, their genomes contributed valuable data to address the longstanding debate about whether DPANN indeed form a monophyletic clade or instead represent an artefactual assemblage of phylogenetically disparate lineages^11–13,26–31,82,83^. In our analyses, which were based on a broader taxon sampling, we not only attempted to account for the effect of compositional biases but also developed a new method for detecting potential HGTs among marker proteins. Our results, based on markers least affected by putative HGTs, consistently recovered clanhood of all DPANN lineages, such that DPANN monophyly (i.e. monophyly of DPANN Cluster 1, Undinarchaeota and DPANN Cluster 2) solely depends on the placement of the root. Notably, in analyses based on phylogenetic markers that had indications for extensive interlineage HGT, Nanoarchaeota and Nanohaloarchaeota group with their respective host clades belonging to the Crenarchaeota and Halobacteria, respectively. This finding indicates that the previous grouping of certain DPANN lineages with Euryarchaeota^28,82^ to some degree is the a result of conflicting signals among commonly used marker proteins (e.g. including those used by PhyloSift^48^ and GTDB^49^), many of which seem unsuited for inferring a reliable tree of life. Finally, our analyses robustly excluded the root from a clade consisting of Euryarchaeota, TACK and Asgard lineages, strengthening the hypothesis that some or all of the DPANN superphylum diverged early from the archaeal tree^27^. In turn, further exploration of DPANN promises to shed light on their roles in biogeochemical cycles and food webs as well as the early evolution of cellular life and symbiosis.

## Methods

### Reconstructing MAGs of Undinarchaeota

We searched for Undinarchaeota-related reads in the Sequence Read Archive (SRA) for public metagenomes using SingleM [https://github.com/wwood/singlem]. Thereby, we found 37 metagenomes containing reads assigned to Undinarchaeota (Supplementary Data 1). Genomes from these 37 metagenomes were recovered by assembly with megahit (v1.1.3)^84^, mapping raw reads from each sample to its corresponding assembly using BamM (v1.7.3) (https://github.com/Ecogenomics/BamM) which uses BWA ‘mem’ (v0.7.15)^85^ with default parameters. Binning was carried out with MetaBAT (v2.11.1)^86^ using default parameters which allowed the reconstruction of six MAGs. The quality of these six as well as the six previously published Undinarchaeota MAGs was assessed using CheckM (v1.0.7)^87^. Additionally, the CheckM marker set was screened for markers that were absent in all 12 Undinarchaeota MAGs. Thereby, we identified seven markers (PF01287, PF01667, PF01912, PF01982, PF04127, TIGR00432 and TIGR01213) that were not only absent in Undinarchaeota but also often in other DPANN archaea (Supplementary Discussion). As the inclusion of these markers might underestimate the completeness scores, we also used CheckM excluding these markers. Both results are summarized in Table 1.

### Contamination screening

Contigs from all 12 Undinarchaeota MAGs were manually investigated for signs of contamination by looking for an abnormal GC-content (~10% difference in average GC-content) and/or taxonomic composition. To determine the taxonomic composition, the protein files of each genome (see section below) were searched against the NCBI-non-redundant (nr) database (downloaded in November 2018) using DIAMOND v0.9.22.123 (settings: –more-sensitive –e-value 1e-10 –seq 100 –no-self-hits –taxonmap prot.accession2taxid.gz)^88^. The best hit for each protein was selected based on the highest e-value and bit-score. Subsequently, the obtained NCBI taxonomy identifier was merged with the taxonomic string information provided by NCBI using a mapping file generated in January 2019 using ncbitax2lin [https://github.com/zyxue/ncbitax2lin]. Contigs with an “abnormal” taxonomic distribution (>90% of proteins on a contig assigned to a non-archaeal or non-DPANN lineage with a percentage identity above 80%) were either removed or confirmed by phylogenetic analyses of single gene trees (see Supplementary Discussion).

Notably, several Undinarchaeota MAGs appeared to encode a short genomic region similar to an archaeal fosmid sequence deposited at NCBI and referred to as uncultured marine group II/III euryarchaeote KM3_51_D01 [https://www.ncbi.nlm.nih.gov/nuccore/663520902]. In particular, proteins encoded in this region shared about 84% ANI to protein homologs found on this fosmid. To investigate whether our MAGs may be contaminated by contigs of members of the MG-II/III archaea (Pontarchaea/Poseidoniales), we performed single-protein trees of marker proteins located in this genomic region and queried the ORFs located on fosmid KM3_51_D01 against NCBI-nr. Importantly, our results (i) suggested that this fosmid does not belong to the Pontarchaea/Poseidoniales archaea and should be reclassified as “uncultured archaeon KM3” and (ii) verified, that respective genomic regions in our MAGs are not contaminated. Please see the Supplementary Discussion for further details.

### Generation of a backbone datasets for phylogenetic analyses

*Archaeal backbones*: To generate a representative archaeal reference set, all archaeal genomes were downloaded from NCBI (January 2019). Genomes with a completeness >40% and contamination <25% (determined with CheckM lineage_wf v1.0.11)^87^ were used for an initial phylogenetic analysis using the PhyloSift marker set. In brief, homologs of 34 PhyloSift marker proteins were identified in each genome using the PhyloSift v1.0.1 ‘find’ mode (settings: –besthit)^49^. Subsequently, marker proteins were individually aligned using MAFFT L-INS-i v7.407 (settings: –reorder)^89^ and trimmed using BMGE v1.12 (settings: -m BLOSUM30 -h 0.55)^90^. The concatenated protein alignment was used to reconstruct a phylogenetic tree using IQ-TREE v1.6.7 (settings: -m LG -nt AUTO -bb 1000 -alrt 1000)^50,59,60^. Based on this tree, a sub-selection of 364 archaeal reference genomes was generated (comprising 352 reference archaeal and 12 Undinarchaeota genomes, Supplementary Data 23), which covers the archaeal phylogenetic diversity and still allows to perform computationally intensive phylogenetic analyses needed to resolve the placement of DPANN in the tree of Archaea. Selected genomes were preferentially derived from type strains as well as from known DPANN symbiont-host taxa. Furthermore, representative MAGs and SAGs were selected based on high completeness and low degree of contamination. Based on an initial maximum-likelihood analysis (IQ-TREE, C60 + LG model) including these 364 species, *Zestosphaera tikiterensis* NZ3 seems to represent a lineage of the order Sulfolobales rather than the Desulfurococcales^20^ with high statistical support (i.e. bootstrap of >97%, Supplementary Fig. 11). Furthermore, the placement of “Acidilobales” within the Desulfurococales was highly supported (i.e. bootstrap of 100%, Supplementary Fig. 11), which indicates that Acidilobaceae represents a family level lineage within the Desulfurococcales rather than an order-level lineage^91^. This also corresponds to the taxonomy of the Genome Taxonomy Database (GTDB; https://gtdb.ecogenomic.org). We therefore assigned both *Zestosphaera tikiterensis* NZ3 and Acidilobaceae to the Desulfurococcales in our analyses.

To create a smaller archaeal reference set suitable for Bayesian phylogenies, the 364 archaeal genome set was further subsampled to only include 127 taxa. In particular, at least two members of all major archaeal order-level lineages were selected based on phylogenetic distance and genome quality. For MAGs that were only classified at phylum level (i.e. Woesearchaeota), we selected a representative set of taxa based on the 364 species tree.

*Bacterial backbones*: To generate a bacterial backbone, we downloaded a list of available genomes from NCBI in 2018 [ftp://ftp.ncbi.nlm.nih.gov/genomes/genbank/bacteria/]. This list was parsed to only include type strains or representative genomes. From this list we randomly selected a representative strain per genus. In a second step, we selected 15–30 genomes from candidate phyla from the unparsed list. This initial set of genomes was screened for completeness and contamination in CheckM^87^ and genomes with less than 50% completeness and more than 10% contamination were excluded. We performed initial phylogenies using alignments generated by PhyloSift^49^ using the PhyloSift search, align and name algorithms to find marker proteins. Individual genes were aligned with MAFFT L-INS-i (settings: –reorder)^89^ and trimmed using BMGE (settings: -m BLOSUM30 -h 0.55)^90^. The concatenated protein alignment was used to reconstruct a phylogenetic tree using IQ-TREE v1.6.7 (settings: -m LG -bb 1000 -alrt 1000)^50^. The initial alignments were screened for long-branches and genomes that did not  cluster into the expected position in the tree were removed from the taxa selection resulting in a set of 3020 representative bacterial genomes (a list of the genomes used is provided in Supplementary Data 23).

*Eukaryotic backbones*: 98 eukaryotic genomes and largely complete transcriptomes were selected so as to obtain even sampling across the known major lineages of eukaryotes. Where possible, we selected free-living representatives of clades with a completely sequenced genome; alternatively, we selected the most complete transcriptome from a free-living member of the group (a list of genomes used is provided in Supplementary Data 23).

### Selection of marker proteins for phylogenetic analyses

To accurately place Undinarchaeota in the archaeal tree of life, we identified suitable markers among three different but overlapping marker proteins sets: (a) a set of herein identified potential single-copy, near universal archaeal orthologues and the marker protein sets implemented in GTDB (b) and PhyloSift (c). A list of the individual marker genes can be found in Supplementary Data 4 and 5.

(a) We inferred a set of markers by running the OMA v2.31 standalone algorithm^92^ on a set of 149 archaeal genomes chosen to represent known taxonomic diversity. We inferred single-protein trees for 97 orthologues (OG) that were present in at least 75% of the genomes and manually inspected the resulting trees to identify and remove 15 markers that had undergone gene transfer, resulting in a final set of 82 candidate single-copy orthologs. (b) The GTDB marker set was downloaded February 2019 (release r86 downloaded from [https://gtdb.ecogenomic.org] and is comprised of 122 HMM profiles from the PFAM and TIGRFAM databases. (c) The PhyloSift marker set^49^ includes a list of 38 HMM profiles [https://phylosift.wordpress.com/tutorials/scripts-markers/], however, excluding genes for the phenylalanine synthetase for which we have observed large numbers of HGTs.

The markers from these three approaches overlapped to a certain degree and in total comprised 151 distinct protein families. Whenever available, we extracted HMM profiles from the PFAM, TIGRFAM or arCOG databases and, if not present in existing databases, profiles were build using HMMbuild. To reduce the risk of detecting distant paralogs in our reference genomes when using protein profiles of just 151 markers as queries, we extended our list of profiles by adding all available TIGRFAM profiles from the TIGRFAM database (TIGRFAMs_15.0_HMM.LIB: downloaded Sept 2018). Altogether, this amounted to 4,528 HMM profiles, 151 of which represented our markers of interest from GTDB, OG and PhyloSift (Supplementary Data 4 and 5). The HMM profiles for all of those combined protein profiles were subsequently queried against a protein database consisting of our archaeal, bacterial and eukaryotic backbone dataset by using a custom script that uses the hmmsearch [options] <seqdb> <hmmfile> algorithm)^93^: hmmsearchTable All_Genomes.faa arc152_inclTIGR_v2.hmm 40 -E 1e-5 > HMMscan_Output_e5.

### Assessment of markers for suitability in concatenations

Subsequently, we extracted archaeal, bacterial and eukaryotic homologs assigned to the 151 marker protein profiles. The output was parsed to only include the best hit based on the e-value and bit-score. Afterwards, the 151 nonredundant marker proteins of interest were extracted from the larger database and individually aligned using MAFFT L-INS-i (settings: –reorder)^89^ and trimmed using BMGE (settings: -t AA -m BLOSUM30 -h 0.55)^90^. Subsequently, we inferred single-protein phylogenies with IQ-tree (settings: -m LG + G -bb 1000 -wbtl -bnni)^50^ for each of these 151 marker protein families, including homologs identified in the corresponding reference genomes (i.e. 364 archaea, 3020 bacteria and 98 eukaryotes (see details above). Marker proteins in which archaea and eukaryotes were not monophyletic (i.e. archaeal/eukaryotic lineages were paraphyletic with some lineages emerging from within Bacteria), were excluded from any further analysis (see also Supplementary Data 4 and 5).

Next, we used a custom python script (count_sister_taxa.py; https://github.com/Tancata/phylo/blob/master/count_sister_taxa.py) to rank each marker protein phylogeny based on the extent to which it resolved monophyletic clades of well-established archaeal phylum- or order-level lineages. In brief, the script counts for each tree, how many times a certain taxon does not group with its expected taxonomic clade across all bootstrap trees, thus representing so-called splits. Splits (which may arise through HGT or phylogenetic artefacts) are counted for each taxon across all protein phylogenies and provide the basis for ranking the different markers according to their reliability. Here, we counted both the total number of splits as well as the splits normalized to the total species count. Note, monophyly was assessed on the level of the following archaeal clades: Geothermarchaeota, Halobacteria, Methanonatronarchaeales, Methanomicrobiales, Methanosarcinales, Methanocellales, Methanophagales, Archaeoglobales, Thermoplasmatales, Acidiprofundales, Methanomassiliicoccales, Poseidoniales, Thermoplasmata (not assigned at order-level), Pontarchaea, Undinarchaeota, Woesearchaeota, Pacearchaeota, NovelDPANN_1 (UAP1), Parvarchaeota, Nanohaloarchaeota, Aenigmarchaeota, Diapherotrites, Huberarchaeota, Micrarchaeota, Altiarchaeota, Methanopyrales, Methanobacteriales, Methanococcales, Desulfurococcales, Sulfolobales, Thermoproteales, Marsarchaeota, Thermococcales, Theionarchaea, Methanofastidiosa, Hadesarchaea, Persephonarchaea, Odinarchaeota, Verstraetearchaeota, Thorarchaeota, Lokiarchaeota, Heimdallarchaeota, Bathyarchaeota, Thaumarchaeota, Korarchaeota, Aigarchaeota, Geoarchaeales, Hydrothermarchaeota and Nanoarchaeota. The number of split phylogenetic clusters (in percentage) as well as the total number of splits normalized by the total number of species within each tree were used as criteria to rank and define the 25, 50 and 75% top as well as 25% and 50% lowest ranking marker proteins (Supplementary Data 4 and 5). Finally, the 50% top-ranking single-protein trees were manually inspected for signs of contamination or paralogues that were then manually removed from the marker protein sequences. After manual cleaning, the marker proteins were aligned using MAFFT L-INS-i^89^ and trimmed using BMGE^90^ as described above. The single proteins were concatenated using catfasta2phyml.pl (https://github.com/nylander/catfasta2phyml). Note that all single-protein phylogenies as well as the individual protein files and alignments have been deposited in a Zenodo repository (10.5281/zenodo.3839790)^94^.

### Phylogenetic analyses for the species trees

To confirm the placement of Undinarchaeota, several different phylogenetic trees were generated (all trees are summarized in Supplementary Table 6 and shown in full in Supplementary Figs. 8–58):

(I) Using different subsets of marker proteins for maximum-likelihood phylogenies: The alignment for the concatenated 25%, 50% and 75% top as well as 25% and 50% lowest ranking marker proteins were generated for the 364 as well as 127 taxa set and trimmed with BMGE (settings: -m BLOSUM30 -h 0.55)^90^. Phylogenetic trees were generated using IQ-TREE (v1.6.7, settings: -m LG + C60 + F + R -bb 1000 -alrt 1000)^50^.
(II) Stationary trimming for maximum-likelihood phylogenies: The concatenated alignment for the 50% top-ranking marker proteins was aligned with MAFFT L-INS-i^89^ and trimmed with BMGE^90^ using stationary trimming (options: -s FAST -h 0:1 -g 1) to remove compositional heterogenous sites from both the 127 and 364 taxa set. Trees were generated using IQ-TREE^50^(settings: -m LG + C60 + F + R -bb 1000 -alrt 1000).
(III) Data recoding for maximum-likelihood phylogenies: The concatenated alignment for the 50% top-ranking marker proteins (alignment: MAFFT L-INS-i^89^, trimming: BMGE^90^) was recoded into four character-states (SR4 recoding^95^; i.e. data simplification from 20 to four character states) to reduce compositional heterogeneity both for the 127 and 364 taxa set using a custom script (Recode_aa.py provided in our data repository). The states used were the following: A,G,N,P,S,T = A; C,H,W,Y = C; D,E,K,Q,R = G and F,I,L,M,V = T. Phylogenetic trees were generated using IQ-TREE (settings: -m C60SR4 -bb 1000 -alrt 1000).
(IV) Removing fast-evolving sites for maximum-likelihood phylogenies: SlowFaster v1^96^ was used on the concatenated alignment for the 50% top-ranking marker proteins (alignment: MAFFT L-INS-i^89^, trimming: BMGE^90^; settings see above) to remove fast-evolving sites both from the 127 and 364 taxa set. Sites were removed in a stepwise manner removing 10, 20, 30 and 40% of the fastest evolving sites. Phylogenetic trees were generated using IQ-TREE^50^ (settings: -m LG + C60 + F + R -bb 1000 -alrt 1000).
(V) Removing heterogenous sites for maximum-likelihood phylogenies: Alignment_pruner.pl (https://github.com/novigit/davinciCode/blob/master/perl) was used to remove heterogenous sites in a stepwise manner from the concatenated alignment for the 50% top-ranking marker proteins (alignment: MAFFT L-INS-i^89^, trimming: BMGE^90^; settings see above) both from the 127 and 364 taxa set. Sites were removed in a stepwise manner removing 10, 20, 30 and 40% of the most heterogeneous sites and the trees were generated using IQ-TREE^50^ (settings: -m LG + C60 + F + R -bb 1000 -alrt 1000).
(VI) Using published marker gene sets for maximum-likelihood phylogenies: Several alternative marker sets were used to confirm the tree topologies. These include the PhyloSift marker set^49^ using the default alignment with hmmalign^97^ or MAFFT L-INS-i^89^, the 122 archaeal GTDB marker set^47^ using FastTree v2.1.10^98^ (settings: WAG, LG) or IQ-TREE^50^ (settings: LG + C60 + F + R), the RP14 marker set^9^ and the universal 48 marker set^5^. Please note that the PhyloSift^49^, 122 GTDB marker gene set^47^ and RP14 marker protein^9^ set were subjected to phylogenetic analyses before defining the 364 taxa set and include slightly less taxa (356).
(VII) Using different subsets of marker proteins for Bayesian phylogenies: The concatenated alignment for the 25% and 50% top-ranking marker proteins (alignment: MAFFT L-INS-i^89^, trimming: BMGE^90^ and the 127 taxa set were used for Bayesian inferences using PhyloBayes-MPI v1.8^51^ (settings: -cat -gtr -x 10 -1 -dgam 4). In particular, for each marker protein family, four parallel chains were run until convergence was reached, unless stated otherwise (maxdiff < 0.3; settings: bpcomp -x 25%_burnin chain1 chain2 chain3 chain4). Additionally, we checked for the minimum effective size using tracecomp (minimum effective size > 50; settings: -x 25%_burnin chain1 chain2 chain3 chain4).
(VIII) Rooting maximum-likelihood phylogenetic trees using nonreversible models: To root our phylogenetic trees, we analyzed the concatenated alignment based on the 50% top-ranking marker proteins using a single partition model and a multiple partition nonreversible model. These analyses were performed on the 127 and 364 taxa set using the full alignments and the recoded alignments in which 20% or 40% of the most heterogeneous sites were removed. Trees were generated with an updated version of IQ-TREE v2^58^ (while all previously mentioned phylogenies were generated with IQ-TREE v1)^50^. The single partition models were run with the following settings: -mset LG -bb 1000 -alrt 1000 -m MFP + MERGE, followed by –model-joint NONREV –min-freq 0.001 -nparam 10 -optfromgiven -bb 1000 -alrt 1000. The multiple partition model was run with these settings: -model-joint NONREV –min-freq 0.001 -nparam 10 -optfromgiven -bb 1000 -alrt 1000. Additionally, we used minimal ancestor deviation (MAD) rooting to determine the root position^99^.
(IX) Rooting maximum-likelihood phylogenetic trees using bacteria as an outgroup: To root our phylogenetic trees with bacteria as an outgroup we added 88 bacterial genomes to our 127 archaeal taxa selection. These 88 bacterial genomes were selected from the bacterial backbone tree by covering a broad diversity and including at least one to three genomes per phylum. However, to minimize LBA artefacts, we did not select members of the CPR^100^ as well as other symbionts or thermophiles, which often emerge on long branches in phylogenetic trees^101,102^. Next, we identified homologs of 48 previously described universal marker proteins^5^ in each genome, individually aligned proteins using MAFFT L-INS-i^89^ (settings: –reorder) and trimmed using BMGE (settings: -t AA -m BLOSUM30 -h 0.55)^90^. Single proteins were concatenated using catfasta2phyml.pl [https://github.com/nylander/catfasta2phyml] and a phylogenetic tree was generated using IQ-TREE (settings: -m LG + C60 + F + R -bb 1000 -alrt 1000).

### Phylogenetic analyses of core Undinarchaeota protein set

A set of 520 proteins for Undinarchaeota were selected based on arCOGs detected in at least three or more Undinarchaeota genomes with an e-value greater than 1e-20 (a raw count table for all arCOGs provided in Supplementary Data 9; a description of the annotations is described below). Next, these 520 arCOGs were queried against a protein database of 364 archaeal, 3020 bacterial and 98 eukaryotic genomes using PSI-BLAST (settings: -e-value 1e-20 -show_gis -outfmt 6 -max_target_seqs 1000 -dbsize 100000000 -comp_based_stats F -seg no) against the arCOG database (version from 2014)^103^. Protein alignments were generated for each individual protein family using MAFFT L-INS-i^89^ (protein alignments with <1000 sequences, settings: –reorder) or MAFFT (alignments with >1000 sequences, settings: –reorder). Afterwards, all alignments were trimmed using BMGE^90^ (settings: -t AA -m BLOSUM30 -h 0.55). Phylogenetic trees were generated using IQ-TREE^50^ (settings: -m LG + G -wbtl -bb 1000 -bnni). To search for HGT events, gene trees were analyzed using the script count_sister_taxa.py [https://github.com/Tancata/phylo/blob/master/count_sister_taxa.py] by providing the ML tree and the bootstrap files as input. For each clade in the maximum-likelihood tree to which a single taxonomic label (see clade of interest below) could be assigned, this script calculates the relative frequencies with which all other clades in the tree were recovered as the closest sister-group, averaging over a sample of 1000 bootstrap trees. One subtlety of the approach is that an archaeal clade cannot be sister to itself, if these two sister lineages are both monophyletic, because they would be considered as a single clade in the analysis. If the sister lineage is itself taxonomically mixed, the relative frequencies of each of the component taxa were augmented proportionally. Cases in which homologs of Undinarchaeota formed a sister lineage to non-DPANN, were visually confirmed and all hits for Undinarchaeota are listed in Supplementary Data 20–22. In particular, we reported for each clade or, if a clade was split, for each sub-clade of interest the closest sister-lineage whenever the normalized occurrence was above a threshold of 0.3.

The clades of interest were: Geothermarchaeota, Halobacteria, Methanonatronarchaeales, Methanomicrobiales, Methanosarcinales, Methanocellales, Methanophagales, Archaeoglobales, Thermoplasmatales, Acidiprofundales, Methanomassiliicoccales, Poseidoniales, Thermoplasmata (not assigned at order-level), Pontarchaea, Undinarchaeota, Woesearchaeota, Pacearchaeota, NovelDPANN_1 (UAP1), Parvarchaeota, Nanohaloarchaeota, Aenigmarchaeota, Diapherotrites, Huberarchaeota, Micrarchaeota, Altiarchaeota, Methanopyrales, Methanobacteriales, Methanococcales, Desulfurococcales, Sulfolobales, Thermoproteales, Marsarchaeota, Thermococcales, Theionarchaea, Methanofastidiosa, Hadesarchaea, Persephonarchaea, Odinarchaeota, Verstraetearchaeota, Thorarchaeota, Lokiarchaeota, Heimdallarchaeota, Bathyarchaeota, Thaumarchaeota, Korarchaeota, Aigarchaeota, Geoarchaeales, Hydrothermarchaeota and Nanoarchaeota).

Subsequently, we counted for each clade/sub-clade of interest, how many times a certain sisterhood relationship was observed as compared to the total amount of sisterhood relationships observed for that clade and plotted this in Fig. 4. Please see our data repository for raw files and scripts used for plotting in the Folder: 3_Scripts/3_R_Table_Figures/2_HGT_analyses^94^.

### Phylogenetic analyses of 16S and 23S rRNA genes

16S and 23S rRNA gene sequences were identified in all archaeal genomes using Barrnap v0.9^104^ (settings: –kingdom arc –e-value 1e-20; [https://github.com/tseemann/barrnap]. Partial sequences and sequences shorter than 400 bp were removed prior to extracting the sequences using bedtools v2.26.0^105^. Subsequently, sequences were individually aligned using MAFFT L-INS-i^89^ and trimmed using TrimAL^106^(v1.2rev59, -automated1 setting) or BMGE^90^ (settings: -t DNA -m DNAPAM100:2 -h 0.55) and concatenated (https://github.com/nylander/catfasta2phyml). 16S and 23S rRNA gene homologs located on the same contig were concatenated whenever more than one copy of the 16S or 23S rRNA gene was encoded by a genome. Alignment_pruner.pl [https://github.com/novigit/davinciCode/blob/master/perl] was used to remove the 10% most heterogenous sites from the alignment. After running an initial tree with all 16S-23S rRNA gene sequences using IQ-TREE, we confirmed that MAGs with multiple copies clustered together and removed all but one representative copy for the following tree. Additionally, we removed 16S and 23S rRNA homologs of the genome-reduced Huberarchaeota to avoid potential long-branch attraction artefacts. A phylogenetic tree was generated with IQ-TREE for the full-length alignment trimmed with TrimAL^106^ (setting: -automated1), BMGE^90^ (settings: -t DNA -m DNAPAM100:2 -h 0.55) as well as the 10% pruned TrimAL alignment (IQ-TREE; settings: -m GTR + G -bb 1000 -alrt 1000)^50^. The trees are provided in Supplementary Figures 3–5.

### Phylogenetic analyses of the RuBisCO gene

RuBisCO protein sequences were identified by their arCOG ID (arCOG04443), which was identified in all Undinarchaeota as described in section ‘Gene calling and annotation’ (the raw annotations are provided in Supplementary Data 7). Subsequently, we extracted the sequences for the RuBisCO protein from Undinarchaeota and combined them with an unmasked alignment generated in a previous study^68^ using mafft_align^89^ with the –add option. The alignment was trimmed using BMGE (-g 0.5 -b 1 -m BLOSUM30 -h 0.55)^90^ and a tree generated using IQ-TREE (-m LG + G -wbtl -bb 1000 -bnni). The conservation of the catalytic site was compared in reference to *Synechococcus elongatus* PCC_6301^67,68^. The tree is provided in Supplementary Fig. 61. The logo for the catalytic site was generated with logomaker v0.8 in python v2.7.15^107^.

### Phylogenetic analyses of the archaeal DNA primase

To compare the phylogenetic history of the canonical and the fused PriL and PriS subunits of the primase we extracted arCOGs corresponding to the two subunits (arCOG04110 for PriS and arCOG03013 for PriL, respectively) from the 364 reference archaeal genomes (Supplementary Data 9). To determine the conserved domains for PriS and PriL in both the fused and canonical proteins we uploaded the sequences to the Conserved Domain Search Service (CD-Search) web-based tool using default settings (Supplementary Data 11). The start and end positions provided by CD-Search were used to split the fused version of the primase into “unfused” PriS and PriL subunits using bedtools getfasta. The sequences for the canonical PriS and PriL as well as split PriS and PriL were combined and aligned using MAFFT L-INS-i^89^(option –reorder) and trimmed using TrimAL^106^ (-gappyout). A phylogenetic tree was generated with IQ-TREE (-m LG + F + C10 -bb 1000 -alrt 1000)^50^. The tree is provided in Supplementary Fig. 59.

### Gene calling and annotation

We annotated the 12 MAGs of Undinarchaeota (six from this study) as well as our set of 352 archaeal reference genomes. Gene calling was performed using Prokka^108^ (v1.14, settings: –kingdom Archaea –addgenes –increment 10 –compliant –centre UU –norrna –notrna). For further functional annotation, the generated protein files were compared against several databases, including the arCOGs (version from 2014)^103^, the KO profiles from the KEGG Automatic Annotation Server^109^ (KAAS; downloaded April 2019), the Pfam database^110^ (Release 31.0), the TIGRFAM database^111^ (Release 15.0), the Carbohydrate-Active enZymes (CAZy) database^112^ (downloaded from dbCAN2 in September 2019), the MEROPs database^113^ (Release 12.0), the Transporter Classification Database^114^ (TCDB; downloaded in November 2018), the hydrogenase database^115^ (HydDB; downloaded in November 2018) and NCBI_nr (downloaded in November 2018). Additionally, all proteins were scanned for protein domains using InterProScan (v5.29-68.0; settings: –iprlookup –goterms)^116^.

Individual database searches were conducted as described in the following section. ArCOGs were assigned using PSI-BLAST v2.7.1 + (settings: -e-value 1e-4 -show_gis -outfmt 6 -max_target_seqs 1000 -dbsize 100000000 -comp_based_stats F -seg no)^117^. KOs as well as PFAMs, TIGRFAMs and CAZymes were identified in all archaeal genomes using hmmsearch v3.1b2^97^ (settings: -E 1e-4). The Merops database was searched using BLASTp v2.7.1 (settings: -outfmt 6, -evalue 1e-20). For all database searches the best hit for each protein was selected based on the highest e-value and bitscore and all results are summarized for Undinarchaeota in Supplementary Data 7 and a count table is provided in Supplementary Data 9. For InterProScan we report multiple hits corresponding to the individual domains of a protein using a custom script (parse_IPRdomains_vs2_GO_2.py). Additionally, tRNA genes were identified from contigs of Undinarchaeota and all archaeal reference genomes using tRNAscan-SE v2.0^118^ and the results are summarized in Supplementary Data 10.

Proteins potentially involved in cell–cell interactions (Supplementary Data 16, some of which were reported in Castelle et al.^14^) were separately screened with HHpred^119^ and Phyre2^120^. HHpred was run on a local server with the HH-suite3 standalone v3.1.0 tool by first running hhblits using the uniclust30_2018_08 database (settings: -E 1E-03, -oa3m). The alignment file in a3m format was used as an input for hhsearch against the pdb70 database (settings: -p 20 -Z 250 -loc -z 1 -b 1 -B 250 -ssm 2 -sc 1 -seq 1 -dbstrlen 10000 -norealign -maxres 32000 -contxt context_data.crf -blasttab). Protein homology was investigated using Phyre2 by using the batch upload function of the web-version [http://www.sbg.bio.ic.ac.uk/~phyre2/html/page.cgi?id=index]. The results are shown in Supplementary Data 18 and 19.

### Metabolic comparisons

Results from the gene calling and annotation described above (Supplementary Data 7) were used as basis for comparative genome analyses and metabolic comparisons. For simplicity we reported gene presence/absence patterns on class level whenever possible, while DPANN and most taxa without cultured representatives were defined at the phylum level. First, the occurrence of each individual gene found across each MAG/reference genome was counted in R (v3.5.0). This count table provided the basis for summary tables generated using the ddply function of the plyr package (v1.8.4). The results of these analyses are summarized in Supplementary Data 9 and 12. To plot the heatmap shown in Fig. 3, the count table was first transferred to a presence/absence matrix and the ddply function was used to summarize the counts across each phylogenetic cluster. The data was visualized as a heatmap using the ggplot function with geom_tile and facet_wrap of the ggplot2 package v3.0.0. A table summarizing the gene IDs used can be found in Supplementary Data 24. The heatmap was manually merged with the collapsed tree in Inkscape v0.91.

### Average amino-acid identity

The average amino-acid identity (AAI) across all archaeal reference genomes and the 12 Undinarchaeota MAGs was calculated using comparem v0.0.23 (settings: aai_wf)^121^. The output was summarized in R (v3.5.0) using the packages reshape2 (v1.4.3), plyr (v1.8.4) and dplyr (v0.7.6) generating the Supplementary Data 3 and ggplot2 (v3.0.0) and ggpubr (v0.2) to plot the data.

### Undinarchaeota co-proportionality analysis

37 SRA metagenomes (Supplementary Data 1) containing reads assigned to Undinarchaeota were pseudo-aligned to a genome dataset consisting of 6,890 GTDB r89 archaeal and bacterial genus-dereplicated genomes (GTDB; [https://gtdb.ecogenomic.org]) and 12 Undinarchaeota MAGs using the software Kallisto v.0.44.0 with the default k-mer size (31 bp)^122^. To reduce the number of false positive genomes, the pseudo-alignment results were subjected to three filtering criteria: (i) the ratio of the observed genome coverage with the expected genome coverage must be greater than 0.3. (ii) The observed extent of genome coverage by the mapped reads must be greater than 0.1. (iii) The number of mapped reads must be greater than 10^122,123^. The subset of genomes that fulfilled the three criteria was used to create a reference database for read mapping of the 37 SRA metagenomes via bamM using default parameters [https://github.com/Ecogenomics/BamM]. Relative abundances (Ar) were normalized based on the total read counts, i.e. the maximum number of reads of all metagenomes (Nm) divided by the sample read count (Ns), multiplied by number of reads of that particular sample that mapped to the genome (r), and the average read length (l), divided by the genome size (g) (Ar=Nm/Ns * (r*l/g))^25,80^. Proportionality was analyzed using the R package propR v 4.2.6^80^ based on normalized relative abundances, followed by the centered log-ratio transformation. Only metagenomes containing a maximum UAP2 normalized relative abundance above 1.0 were included in this step (that resulted in 27 metagenomes involved in calculating proportionality). Finally, genomes that were proportional (ρ ≥ 0.9) to more than three Undinarchaeota MAGs were identified as Undinarchaeota co-correlated.

### Reporting summary

Further information on research design is available in the Nature Research Reporting Summary linked to this article.

## Supplementary information

Supplementary Information

Peer Review

Reporting summary

Description of Additional Supplementary Files

Supplementary Data 1–24

## Data Availability

All datasets generated and/or analysed during this study are available in our data repository at Zenodo [10.5281/zenodo.3839790]. Furthermore, MAGs can additionally be accessed at NCBI under the BioProject ID PRJNA609027 (MAG-specific accession numbers can be found in Supplementary Data 1). Furthermore, additional supplementary files including contigs and protein files for 12 Undinarchaeota MAGs, the 352 reference genomes as well as phylogenies for the species and single gene tree analyses (i.e. protein files, alignments and treefiles) (Supplementary Figs. 6–56) have been deposited at our repository Zenodo [10.5281/zenodo.3839790]. Public databases used in this study are the following: The GTDB marker set downloaded February 2019 (release r86 downloaded from [https://gtdb.ecogenomic.org], the arCOG database (version from 2014) downloaded from [ftp://ftp.ncbi.nih.gov/pub/wolf/COGs/arCOG/], the KO profiles downloaded from the KEGG Automatic Annotation Server in 2019 [https://www.genome.jp/tools/kofamkoala/], the Pfam database (Release 31.0) [ftp://ftp.ebi.ac.uk/pub/databases/Pfam/releases/], the TIGRFAM database (Release 15.0) [ftp://ftp.jcvi.org/pub/data/TIGRFAMs/], the Carbohydrate-Active enZymes (CAZy) database downloaded from dbCAN2 in September 2019 [http://bcb.unl.edu/dbCAN2/download/], the MEROPs database (Release 12.0) [https://www.ebi.ac.uk/merops/download_list.shtml], the Transporter Classification Database(TCDB) downloaded in November 2018 [http://www.tcdb.org/download.php], the hydrogenase database (HydDB) downloaded in November 2018 [https://services.birc.au.dk/hyddb/browser/] and NCBI_nr downloaded in November 2018 [ftp://ftp.ncbi.nlm.nih.gov/blast/db/].

## Code availability

All workflows for the annotations and phylogenies as well as custom python-, perl- and R-scripts to analyze tree files, parse data and generate figures),  have been deposited at Zenodo with the [10.5281/zenodo.3839790]. Additionally, we used to following published custom code: BamM (v1.7.3) [https://github.com/Ecogenomics/BamM], ncbitax2lin [https://github.com/zyxue/ncbitax2lin], count_sister_taxa.py; [https://github.com/Tancata/phylo/blob/master/count_sister_taxa.py, catfasta2phyml.pl [https://github.com/nylander/catfasta2phyml] and Alignment_pruner.pl [https://github.com/novigit/davinciCode/blob/master/perl].
