# Supplementary material for: Undinarchaeota illuminate DPANN phylogeny and the impact of gene transfer on archaeal evolution
Source: Nat Commun. 2020 Aug 7;11:3939. doi: 10.1038/s41467-020-17408-w (PMC7414124; doi:10.1038/s41467-020-17408-w)
Supplement: Supplementary file 3 — Reporting summary [file 41467_2020_17408_MOESM3_ESM.pdf]

nature research

Corresponding author(s): Nina Dombrowski, Tom A. Williams, Jiarui Sun, Benjamin J. Woodcroft, Jun-Hoe Lee, Bui Quang Minh, Christian Rinke, Anja Spang

Last updated by author(s): Mar 26, 2020

Reporting Summary

Nature Research wishes to improve the reproducibility of the work that we publish. This form provides structure for consistency and transparency in reporting. For further information on Nature Research policies, see Authors & Referees and the Editorial Policy Checklist .

Statistics

For all statistical analyses, confirm that the following items are present in the figure legend, table legend, main text, or Methods section.

n/a	Confirmed	
☒	☐	The exact sample size (*n*) for each experimental group/condition, given as a discrete number and unit of measurement
☒	☐	A statement on whether measurements were taken from distinct samples or whether the same sample was measured repeatedly
☒	☐	The statistical test(s) used AND whether they are one- or two-sided<br>*Only common tests should be described solely by name; describe more complex techniques in the Methods section.*
☒	☐	A description of all covariates tested
☒	☐	A description of any assumptions or corrections, such as tests of normality and adjustment for multiple comparisons
☒	☐	A full description of the statistical parameters including central tendency (e.g. means) or other basic estimates (e.g. regression coefficient) AND variation (e.g. standard deviation) or associated estimates of uncertainty (e.g. confidence intervals)
☒	☐	For null hypothesis testing, the test statistic (e.g. *F*, *t*, *r*) with confidence intervals, effect sizes, degrees of freedom and *P* value noted<br>*Give P values as exact values whenever suitable.*
☒	☐	For Bayesian analysis, information on the choice of priors and Markov chain Monte Carlo settings
☒	☐	For hierarchical and complex designs, identification of the appropriate level for tests and full reporting of outcomes
☒	☐	Estimates of effect sizes (e.g. Cohen's *d*, Pearson's *r*), indicating how they were calculated

Our web collection on statistics for biologists contains articles on many of the points above.

Software and code

Policy information about availability of computer code

Data collection

Data was collected from NCBI and treated with the following software programs: megahit (v1.1.3), BamM (v1.7.3), BWA 'mem' (v0.7.15), MetaBAT (v2.11.1)

Data analysis

All workflows for the annotations and phylogenies as well as custom python and R-scripts (to analyze tree files), perl code (to parse data) and R codes (to parse the data and generate figures), have been deposited at Zenodo with the [doi: 10.5281/zenodo.3839790].

The version numbers of used softwares are: CheckM (v1.0.7), diamond v0.9.22.123, PhyloSift v1.0.1, mafft-linsi v7.407, BMGE v1.12, IQ-TREE v1.6.7 and IQ-TREE v2 (v2 was used for testing the non-reversible model, v1 was used for all other phylogenies), PhyloBayes-MPI v1.8, FastTree v2.1.10, SlowFaster v1, HMMER v3.1b2 (including hmmsearch and hmmalign), , barrnap v0.9, TrimAL v1.2rev59, Conserved Domain Search Service (CD-Search) web-based tool, prokka v1.14, InterProScan v5.29-68.0, psiblast v2.7.1+, blastp (v2.7.1), DIAMOND v0.9.22.123, OMA v2.3.1, tRNAscan-SE v2.0, Phyre2 web-server, HH-suite3 standalone v3.1.0, R (v3.5.0), R package ddply v 1.8.4, R package ggplot2 v3.0.0, R package reshape2 (v1.4.3), R package plyr v1.8.4, R package ggpubr (v0.2), R package propR v4.2.6, inkscape v0.91, comparem v0.0.23, Kallisto v.0.44.0, bamM ((https://github.com/Ecogenomics/BamM)), logomaker v0.8 in python v2.7.15, Jalview 2.10.5.4, figtree v1.4.4, Illustrator 2018

The version numbers and/or links of used softwares are: BamM (v1.7.3) (https://github.com/Ecogenomics/BamM). SingleM (https://github.com/wwood/singlem), ncbitax2lin (https://github.com/zyxue/ncbitax2lin), count_sister_taxa.py (https://github.com/Tancata/phylo/blob/master/count_sister_taxa.py); catfasta2phyml.pl (https://github.com/nylander/catfasta2phyml); alignment_pruner.pl (https://github.com/novigit/davinciCode/blob/master/perl)

Used databases are the following: The GTDB marker set downloaded February 2019 541 (release r86 downloaded from [https://gtdb.ecogenomic.org], arCOGs (version from 2014) downloaded from[ftp://ftp.ncbi.nih.gov/pub/wolf/COGs/arCOG/], the KO profiles downloaded from the KEGG Automatic Annotation Server in 2019[https://www.genome.jp/tools/kofamkoala/], the Pfam database (Release 31.0)[ftp://ftp.ebi.ac.uk/pub/databases/Pfam/releases/], the TIGRFAM database (Release 15.0)[ftp://ftp.jcvi.org/pub/data/

TIGRFAMs/], the Carbohydrate-Active enZymes (CAZy) database downloaded from dbCAN2 in September 2019[http://bcb.unl.edu/dbCAN2/download/], the MEROPs database (Release 12.0)[https://www.ebi.ac.uk/merops/download_list.shtml], the Transporter Classification Database(TCDB) downloaded in November 2018[http://www.tcdb.org/download.php], the hydrogenase database (HydDB) downloaded in November 2018[https://services.birc.au.dk/hyddb/browser/] and NCBI_nr downloaded in November 2018[ftp://ftp.ncbi.nlm.nih.gov/blast/db/].

For manuscripts utilizing custom algorithms or software that are central to the research but not yet described in published literature, software must be made available to editors/reviewers. We strongly encourage code deposition in a community repository (e.g. GitHub). See the Nature Research guidelines for submitting code & software for further information.

Data

Policy information about availability of data

All manuscripts must include a data availability statement. This statement should provide the following information, where applicable:

- Accession codes, unique identifiers, or web links for publicly available datasets
- A list of figures that have associated raw data
- A description of any restrictions on data availability

All data sets generated and/or analysed during this study are available in our data repository at Zenodo [doi: 10.5281/zenodo.3839790]. Furthermore, MAGs can additionally be accessed at NCBI [https://www.ncbi.nlm.nih.gov/] under the BioProject ID PRJNA609027 (MAG-specific accession numbers can be found in Supplementary Data 1). Furthermore, additional supplementary files including contigs and protein files for 12 Undinarchaeota MAGs, the 352 reference genomes as well as phylogenies for the species and single gene tree analyses (i.e. protein files, alignments and treefiles) (Supplementary Figures 6-56) have been deposited at our repository Zenodo [doi: 10.5281/zenodo.3839790].

Public databases used in this study are the following: The GTDB marker set downloaded February 2019 (release r86 downloaded from [https://gtdb.ecogenomic.org], the arCOG database (version from 2014) downloaded from [ftp://ftp.ncbi.nih.gov/pub/wolf/COGs/arCOG/], the KO profiles downloaded from the KEGG Automatic Annotation Server in 2019 [https://www.genome.jp/tools/kofamkoala/], the Pfam database (Release 31.0) [ftp://ftp.ebi.ac.uk/pub/databases/Pfam/releases/], the TIGRFAM database (Release 15.0) [ftp://ftp.jcvi.org/pub/data/TIGRFAMs/], the Carbohydrate-Active enZymes (CAZy) database downloaded from dbCAN2 in September 2019 [http://bcb.unl.edu/dbCAN2/download/], the MEROPs database (Release 12.0) [https://www.ebi.ac.uk/merops/download_list.shtml], the Transporter Classification Database(TCDB) downloaded in November 2018 [http://www.tcdb.org/download.php], the hydrogenase database (HydDB) downloaded in November 2018 [https://services.birc.au.dk/hyddb/browser/] and NCBI_nr downloaded in November 2018 [ftp://ftp.ncbi.nlm.nih.gov/blast/db/].

Field-specific reporting

Please select the one below that is the best fit for your research. If you are not sure, read the appropriate sections before making your selection.

☐ Life sciences ☐ Behavioural & social sciences ☒ Ecological, evolutionary & environmental sciences

For a reference copy of the document with all sections, see nature.com/documents/nr-reporting-summary-flat.pdf

Ecological, evolutionary & environmental sciences study design

All studies must disclose on these points even when the disclosure is negative.

Study description	The Sequence Read Archive (SRA) was mined for public metagenomes containing UAP2 using singleM (https://github.com/wwood/singlem) in order to increase the available genomes. Thereby, we obtained a total of 6 metagenome-assembled genomes (MAGs), which we analyzed together with the six previously published UAP2 MAGs and other archaeal MAGs. These genomes were used to (a) re-asses the placement of UAP2 in the archaeal tree of life, (b) investigate the monophyly of DPANN archaea and (c) investigate the metabolic potential of UAP2 relative to other DPANN archaea, (d) assess the extend of horizontal gene transfer among DPANN and their hosts.
Research sample	All publicly available metagenomes at NCBI were mined for UAP2 MAGs. This selection should hold the largest amount of sequence data available to find more representatives of this group and allowed us to recover 6 MAGs used for further analyses.
Sampling strategy	na
Data collection	na
Timing and spatial scale	na
Data exclusions	na
Reproducibility	na
Randomization	na
Blinding	na

Did the study involve field work? ☐ Yes ☒ No

Reporting for specific materials, systems and methods

We require information from authors about some types of materials, experimental systems and methods used in many studies. Here, indicate whether each material, system or method listed is relevant to your study. If you are not sure if a list item applies to your research, read the appropriate section before selecting a response.

Materials & experimental systems

n/a	Involved in the study
☒	☐ Antibodies
☒	☐ Eukaryotic cell lines
☒	☐ Palaeontology
☒	☐ Animals and other organisms
☒	☐ Human research participants
☒	☐ Clinical data

Methods

n/a	Involved in the study
☒	☐ ChIP-seq
☒	☐ Flow cytometry
☒	☐ MRI-based neuroimaging
