# Supplementary material for: Undinarchaeota illuminate DPANN phylogeny and the impact of gene transfer on archaeal evolution
Source: Nat Commun. 2020 Aug 7;11:3939. doi: 10.1038/s41467-020-17408-w (PMC7414124; doi:10.1038/s41467-020-17408-w)
Supplement: Supplementary file 4 — Description of Additional Supplementary Files [file 41467_2020_17408_MOESM4_ESM.pdf]

Description of Additional Supplementary Files

File Name: Supplementary Data 1
Description: List of Undinarchaeota-positive metagenomes used for the abundance and co-occurrence analyses

File Name: Supplementary Data 2
Description: General genome statistics and information for each metagenomic sample that was used to assemble and bin Undinarchaeota genomes. Includes the original genome name, isolation source of the metagenome, metagenome ID as well as details on sampling procedure (if available). Additionally, the degree genome completeness and contamination was estimated using CheckM. The CheckM results were investigated for marker genes commonly absent in DPANN archaea (see Supplementary Discussion) and CheckM was re-run excluding seven marker proteins. The results of this analysis are shown in parentheses.

File Name: Supplementary Data 3
Description: Amino acid identity values (in %) comparing 352 reference archaeal genomes and 12 Undinarchaeota MAGs. The taxonomy string includes phylum, class, order, family, genus and species names separated by a '-'. If any taxonomy level was not classified it is indicated with a 'none'.

File Name: Supplementary Data 4
Description: Summary of marker sets used for maximum-likelihood phylogenetic analyses using the 364 taxa set. Marker genes were ranked by the number of splits per phylogenetic cluster and total splits normalized by species count (Rank A + Rank B). A split is defined as non-monophyletic major archaeal lineages usually defined at the class level. For example, if Methanomicrobiales were not monophyletic in one of the single gene trees, this was counted as a split group. ArCOG = Archaeal Clusters of Orthologous Genes.

File Name: Supplementary Data 5
Description: Summary of marker sets used for Bayesian and maximum-likelihood phylogenetic analyses using the 127 species taxa set. Marker genes were ranked by the number of splits per phylogenetic cluster and total splits normalized by species count (Rank A + Rank B). A split is defined as non-monophyletic major archaeal lineages usually defined at the class level. For example, if Methanomicrobiales were not monophyletic in one of the single gene trees, this was counted as a split group. ArCOG = Archaeal Clusters of Orthologous Genes.

File Name: Supplementary Data 6
Description: Summary of phylogenetic analyses listing the number of species included in individual trees, the alignment length as well as the method to generate individual phylogenetic trees. The trees were rooted either using the non-reversible (NR) method in IQ-TREE or using minimal ancestor deviation (MAD) rooting that was applied on the unrooted tree file. AI = Ambiguity index (This ratio will be 1 for when two or more root

positions are found). CCV = Clock coefficient of variation. A/M/D: Altiarchaeota/Micrarchaeota/Diapherotrites.

File Name: Supplementary Data 7
Description: Annotation table of Undinarchaeota proteins generated using different databases (for details see the Methods section). ArCOG = Archaeal Clusters of Orthologous Genes. KO = KEGG Orthology. CAZymes = Carbohydrate-Active enZYmes . TCDB = Transporter classification database. HydDB = Hydrogenase database.

File Name: Supplementary Data 8
Description: Annotation table of Undinarchaeota proteins ordered by key pathways and processes of interest.

File Name: Supplementary Data 9
Description: Table listing protein occurrences across major archaeal taxonomic clusters (in %) as well as the total protein counts for individual Undinarchaeota MAGs. List the counts for different database searches including the ArCOG, KEGG, PFAM and TIGRFAM databases (columns Marker_ID and Database). The average occurrence in percent for archaea versus DPANN archaea is shown in Columns BQ and BR, respectively. Number in parentheses: Number of genomes included in each phylogenetic cluster. Orange: Undinarchaeales. Yellow: Naiadarchaeales.

File Name: Supplementary Data 10

Description: List of identified tRNA synthetases across Undinarchaeota MAGs (green) and archaeal reference genomes

File Name: Supplementary Data 11
Description: Primase-specific domains identified by CDD-Search. CDD-Search was run on proteins annotated as primase (arCOG04110 and arCOG03013) for all Undinarchaeota MAGs and archaeal reference genomes. Canonical primase = PriS and PriL subunits are encoded on different genes. Fused primase = PriS and PriL are fused and located in the same gene. Whether a primase was defined as canonical or fused was decided based on the CDD hit search. CDD = Conserved Domains Database.

File Name: Supplementary Data 12
Description: Summary of key metabolic proteins (based on ArCOG, TIGR and PFAM annotations). Ordered based on occurrence in key metabolic pathways and listing protein occurrence across major archaeal taxonomic clusters (%). Additionally, shows the total counts for individual Undinarchaeota MAGs. Number in parentheses: Number of genomes included in each phylogenetic cluster.

File Name: Supplementary Data 13
Description: Count table of identified transporters in Undinarchaeota MAGs. TCDB = Transporter classification database.

File Name: Supplementary Data 14

Description: Count table of identified carbohydrate-active enzymes (CAZymes) in Undinarchaeota MAGs

File Name: Supplementary Data 15
Description: Count table of identified peptidases (based on the MEROPS database) in Undinarchaeota MAGs

File Name: Supplementary Data 16
Description: List of candidate proteins potentially involved in cell-cell interactions (some of these have been
reported in Castelle et al., 2018)

File Name: Supplementary Data 17
Description: Occurrence of cell-cell interaction related IPR domains across archaeal clades (extracted from Supplementary Data 16). Normalized to the total number of proteins present in each genome and presented as the fraction of the total genome (in percent)

File Name: Supplementary Data 18
Description: Hhpred results for selected proteins related to cell-cell interactions. Showing the Top20 highest
ranked scores. Hits related to cell adhesion or extracellular matrix domains are highlighted in green.

File Name: Supplementary Data 19
Description: Phyre2 results for selected proteins related to cell-cell interactions. Showing the Top 20 highest ranked scores. Hits related to cell adhesion or extracellular matrix domains are highlighted in green.

File Name: Supplementary Data 20
Description: Occurrence of ArCOGs belonging to the core Undinarchaeota protein set across the archaeal, bacterial and eukaryotic reference genomes. The occurrence is shown in percent and the total numbers of genomes included in each phylogenetic cluster is shown in parentheses.

File Name: Supplementary Data 21
Description: Core Undinarchaeota protein set used to identify potential HGT events. Shows the raw count table of sister lineages to Undinarchaeota (group of interest) and the normalized_sum_of_occurances that was calculated over 1000 bootstrap files. Note, that the occurrences are summarized across 1000 trees and therefore it is possible that certain positions are not reflected in the final treefile that shows the best tree by maximum likelihood. Therefore, Undinarchaeota-related hits were manually investigated and genes with a normalized occurrence >=0.1 are shown and additionally hits with an occurrence >= 0.3 were manually inspected and details are noted in the comments section. Columns B-D summarize the occurrence of an arcog

across the archaeal (n=364), bacterial (n=3020) and eukaryotic (n=100) genomes in percent.

File Name: Supplementary Data 22
Description: Core Undinarchaeota protein set used to identify potential HGT events. Shows the percentage of identified sister lineages (Sl) for all archaeal clades of interest (CoI). Only genes with a normalized occurrence >= 0.3 were considered to calculate the percentage of all potential gene transfers. The full list of genes and the hits per gene are shown in Supplementary Data 20 and the sister lineages for Undinarchaeota are shown in Supplementary Data 21.

File Name: Supplementary Data 23
Description: List of genomes used in this study. For each genome the NCBI (or JGI) accession number, taxonomic
ID and submission details are listed. Additionally, general genome statistics were determined using CheckM.

File Name: Supplementary Data 24
Description: List of key proteins used to generate heatmaps (Fig. 4 and Supplementary Figs S60, S62 and S63).
For each gene the respective pathway, protein ID, protein description and used identifier are indicated.
