# Supplementary material for: Undinarchaeota illuminate DPANN phylogeny and the impact of gene transfer on archaeal evolution
Source: Nat Commun. 2020 Aug 7;11:3939. doi: 10.1038/s41467-020-17408-w (PMC7414124; doi:10.1038/s41467-020-17408-w)
Supplement: Supplementary file 2 — Peer Review [file 41467_2020_17408_MOESM2_ESM.pdf]

Reviewer #1 (Remarks to the Author):

In this article, Dombrowski and colleagues describe how they obtained 6 good quality (medium to high) genomes from publicly available metagenomes (MAGs) for the "Uncultivated Archaeal Phylum 2" (UAP2) group of archaea. This group is interesting as previously described as deeply-branching within Archaea, in particular in the debated clan of "DPANN. The authors analysed the obtained MAGs together with 6 previously described MAGs of UAP2, and propose a thorough phylogenomic analysis. They provide a robust phylogenetic analysis based on several marker genes sets, taking into account several possible sources of phylogenetic incongruences and artifacts. In order to extract marker genes that are most suitable to build species phylogenies, they propose an original ranking system based on the recovery of largely accepted phylogenetic groups. The controlled phylogenetic reconstructions repeatedly resulted in the positioning of UAP2 as an independent lineage within a DPANN clan. They therefore propose to elevate to the UAP2 at the phylum level under the name "Udinarchaeota". Their phylogenetic proximity and proposed monophyly with the so-called "Cluster 2" within the DPANN clan was proposed, and further supported by shared genomic traits. This study also offers an assessment of the Udinarchaeota metabolic capacities that suggest they are mostly fermentative and might engage in symbiosis, even though hosts could not be clearly identified.

Overall, this study offers important insights into deep Archaeal phylogeny (clanhood of DPANN and positioning of UAP2), while shedding light on a little understood, newly discovered DPANN lineage that is very likely to mostly include symbionts. The article is well-written and clearly reflects a considerable amount of work. The conclusions seem supported by the data and analyses. I also would like to acknowledge the authors for the efforts put in making the extensive datasets and tools used, widely available to the community.

Main comments

- I believe the main text should better reflect the amount of work invested in phylogenetic analyses. It is mostly discussed how taking into account compositional biases showed the robust positioning of Udinarchaeaota as an independent lineage, close to DPANN Cluster 2. Biases in per-site substitution rates were also investigated by removing fast-evolving sites, but the outcome is not discussed in section on lines 179-191. Even though results were consistent throughout sites removal, this should be stated as it demonstrated further robustness in the phylogenetic positioning of Udinarchaeota (and maybe no impact from per-site rate variations?).

- Regarding the phylogenetic approach taken to rank marker genes by phylogenetic incongruence, I'm wondering if the marker genes having the lowest rankings have mostly supported incongruences, or if they just rather suffer from a lack of phylogenetic signal. These would represent very different reasons for their "unsuitability" for inferring species phylogenies. Did the authors look into this systematically? I believe a brief discussion about this would be interesting for many in the field given how widely these gene sets are being used, on top of what is discussed in Supp Mat (lines 89-102).

- On the same note, regarding the detection of horizontal gene transfers in section on line 330 ("Insights into interaction partners"), I believe that it would be more convincing if some clear cases of horizontal gene transfers were to be shown in trees/discussed even briefly in Sup Mat. For instance, could the family discussed on line 356-357 be shown in a tree?

- It would have been interesting to see in how many other lineages the 520 core genes had detected homologs. I guess all core genes don't offer the same power to detect HGT given their taxonomic distribution in other lineages? Maybe could this be added to Table S16 as the number of lineages where the core gene was found? The number of Udinarchaeota genomes harbouring each core gene could also be specified here. By the way, I don't understand why there are more than 520 lines on that table? I can see that each marker appears several times? Does this reflect the different observations from bootstrap trees?

- The authors attempted to search hosts with a technique that I believe which might favour the detection of conserved, shared hosts across Udinarchaeota. Could this be that these species have developed specific interactions with different hosts along their diversification? In which case, could this explain why no HGT route is observable, unlike for other DPANN lineages? What is the author's view on this aspect?

- Line 365-366, it is mentioned that Udinarchaeales could co-occur with some Dehalococcoidales. Could the authors find any signs of potential HGT between Chloroflexi and Udinarchaeota? How likely do the authors think it is that the potentially exchanged genes are not part of the core genes analysed?

Other comments

- Line 149: it is said that the "best-fitting models of sequence evolution" were used, yet it does not seem that this was statistically assessed e.g. using the "–m TEST" option of IQ-Tree. Maybe just say they are among the "most sophisticated models"?

- Line 151: I haven't seen any mention of statistical topology testing? Where did these analyses take place in the study? To test which topology against which?

- Line 476: "We mined…" specify that the authors searched for Udinarchaeota?

- Line 494: To screen for contaminants, it is said that Diamond was used to assess taxonomic origin, but in Sup Mat, it is said line 34 that Blastp was used. So which is it?

- Line 570: I don't understand what is meant by "combining TIGRFAM and GTDB"? What's the goal here? How is it ensured that TIGRFAM families meet the pre-requisites to build species trees (universality + unicity in genomes)? I believe some details are missing here.

- In the Methods, I believe that the numerous supplementary tables should be referred to on more occasions, in order to help the reader find the datasets mentioned along the text. For instance, section starting on line 518: where is the list of genomes used as the "backbone dataset"? Section starting on line 559: refer to the tables S4 and S5? etc…

Figures

- Figure 1: The text in the box is inconsistent with the legend as it is said in the legend: "40% of the most heterog. sites were removed". So which is it?

- Figure 2: The legend could be improved by mentioning what's represented in grey. It seems it corresponds to what's missing from all genomes.

- Figure 3 and Supp Figures 58-61: Indicating the Cluster 2 of DPANN on these figures could help (with brackets?) or instead, just mention in the figures legends that they are above Udinarchaeota within the DPANN rectangle?

- Figure 4: It is confusing, as there is a title to the figure in the legend "Potential HGTs..." and one on the figure: "Phylogenetic relationships of Undin. core proteins". I think the title in the legend is misleading. It's not only showing potential HGTs, but overall phylogenetic relatedness of the genes. Some could be interpreted as HGTs. I'd rather use the title displayed on the figure, that is more reflective of the figure content.

- Table 1. In the table legend, "parentheses" should replace "brackets".

Typos

line 71: “artefacts”, line 82: “artifacts” => pick one?
line 71: “branch”
line 132: “marker”
line 388: “affected”
line 695 “sequences” missing in “with <1000”

Reviewer #2 (Remarks to the Author):

This manuscript makes an impressive contribution to our understanding of DPANN evolution and ecology and is clearly the product of extensive and well performed analysis, even if most of it ended up in the huge supplementary information section. Furthermore, the analysis of HGT and how it affects the phylogeny of currently disputed archaeal groups is highly valuable for the field.
I am not sure why in the age of online only publications manuscripts must be kept so short, and there are sections in the supplementary information, especially those describing gene content, that should have been in the main text, IMHO.

I have only minor points, see below.

"Nanoarchaeota are ectosymbionts of TACK archaea" - it would be better here to mention the specific lineages within TACK or at least spell out TACK for the reader.
Table 1 - strain heterogeneity is not clearly presented
"low ranking makers were highly incongruent" - should be "... markers..."
"clan" and "clanhood" are relatively recent terms in phylogenetics that most readers will be unfamiliar with and should be introduced.

Since the archaeal root cannot be reliably inferred are there synapomorphies supporting DPANN as a clan?

"Nanohaloarchaeota and Halobacteria shared a large amount of analyzed 345 proteins horizontally (12.4% and 7%)" - unclear what the two different % refer to in this sentence.

Either figure 4 is not adequately explained or there are lineages for which there are more proteins that originate from HGT than by shared ancestry (for example the Halobacteria). Such cases have been shown in bacteria (for example the Thermotogales), but not in archaea, so if this is indeed the case for multiple lineages the text should say something about it.

Response letter

Reviewer #1 (Remarks to the Author):

In this article, Dombrowski and colleagues describe how they obtained 6 good quality (medium to high) genomes from publicly available metagenomes (MAGs) for the "Uncultivated Archaeal Phylum 2" (UAP2) group of archaea. This group is interesting as previously described as deeply-branching within Archaea, in particular in the debated clan of "DPANN. The authors analysed the obtained MAGs together with 6 previously described MAGs of UAP2, and propose a thorough phylogenomic analysis. They provide a robust phylogenetic analysis based on several marker genes sets, taking into account several possible sources of phylogenetic incongruences and artifacts. In order to extract marker genes that are most suitable to build species phylogenies, they propose an original ranking system based on the recovery of largely accepted phylogenetic groups. The controlled phylogenetic reconstructions repeatedly resulted in the positioning of UAP2 as an independent lineage within a DPANN clan. They therefore propose to elevate to the UAP2 at the phylum level under the name "Udinarchaeota". Their phylogenetic proximity and proposed monophyly with the so-called "Cluster 2" within the DPANN clan was proposed, and further supported by shared genomic traits. This study also offers an assessment of the Udinarchaeota metabolic capacities that suggest they are mostly fermentative and might engage in symbiosis, even though hosts could not be clearly identified.

Overall, this study offers important insights into deep Archaeal phylogeny (clanhood of DPANN and positioning of UAP2), while shedding light on a little understood, newly discovered DPANN lineage that is very likely to mostly include symbionts. The article is well-written and clearly reflects a considerable amount of work. The conclusions seem supported by the data and analyses. I also would like to acknowledge the authors for the efforts put in making the extensive datasets and tools used, widely available to the community.

Response: thank you very much for this kind and supportive assessment.

Main comments
- I believe the main text should better reflect the amount of work invested in phylogenetic analyses. It is mostly discussed how taking into account compositional biases showed the robust positioning of Udinarchaeaota as an independent lineage, close to DPANN Cluster 2. Biases in per-site substitution rates were also investigated by removing fast-evolving sites, but the outcome is not discussed in section on lines 179-191. Even though results were consistent throughout sites removal, this should be stated as it demonstrated further robustness in the phylogenetic positioning of Udinarchaeota (and maybe no impact from per-site rate variations?).

Response: We agree with the reviewer and have now extended the section on phylogenetic analyses by also mentioning results from per-site substitution rates and by describing in more detail the results of our marker selection analysis (see below). (**see lines 266 and 366-368)**

- Regarding the phylogenetic approach taken to rank marker genes by phylogenetic incongruence, I'm wondering if the marker genes having the lowest rankings have mostly supported incongruences, or if they just rather suffer from a lack of phylogenetic signal. These would represent very different reasons for their "unsuitability" for inferring species phylogenies. Did the authors look into this systematically? I believe a brief discussion about this would be interesting for many in the field given how widely these gene sets are being used, on top of what is discussed in Supp Mat (lines 89-102).

Response: This is an interesting point. Our scoring system weights incongruences by their bootstrap supports: in principle, a low-scoring marker gene could either strongly reject a small number of established clades, or weakly reject a larger number of them. To investigate to what extent poor markers are low-scoring due to lack of phylogenetic signal, we now compared protein and alignment length as well as total bootstrap support differences across the highest and lowest ranked markers (new S-Figure 6a-c). Furthermore, we investigated the phylogenetic relationship and support for the placement of two known symbionts with their hosts to evaluate whether potential HGTs can be highly supported (S-Figure 7a-d). This revealed that, while low scoring markers are overall shorter and have slightly lower overall bootstrap support, they can still recover highly supported clans that are indicative of HGT events. We have added these new results to the main text. (**see lines 152-164).**

- On the same note, regarding the detection of horizontal gene transfers in section on line 330 ("Insights into interaction partners"), I believe that it would be more convincing if some clear cases of horizontal gene transfers were to be shown in trees/discussed even briefly in Sup Mat. For instance, could the family discussed on line 356-357 be shown in a tree?

Response: We agree with the reviewer that a few examples would be nice to illustrate potential HGTs. Marker genes with potential HGTs between known symbiont-host systems are described in **Supplementary Table 4** and corresponding trees can be downloaded from our data repository. In addition, and as suggested, we have now selected three trees (corresponding to two protein families) **(Supplementary Figure 65, panel a-c),** to illustrate potential horizontal evolution among Undinarchaeota and potential partners in the section ("Insights into interaction partners"). (**see line 610 and 619-622).**

- It would have been interesting to see in how many other lineages the 520 core genes had detected homologs. I guess all core genes don't offer the same power to detect HGT given their taxonomic distribution in other lineages? Maybe could this be added to Table S16 as the number of lineages where the core gene was found? The number of Udinarchaeota genomes harbouring each core gene could also be specified here. By the way, I don't understand why there are more than 520 lines on that table? I can see that each marker appears several times? Does this reflect the different observations from bootstrap trees?

Response: This is a good point and we have now added this information to the new Table S20 (lists the presence of all investigated arCOGs across all investigated lineages of interest) and 21 (the latter of which corresponds to the previous Table S16). It seems that the core genes of the Undinarchaeota are widely shared among all archaeal lineages (on average the proteins investigated are present in ~61% archaeal lineages and 310 of the families are present in more than 50% of the archaeal species) suggesting that they represent a good approximation for detecting HGT. In this regard, we also want to mention that while not included in this manuscript, we have in fact repeated this analysis with all arcogs (not only those in at least three Undinarchaeota), which confirmed the patterns we see with this smaller selection of markers.
And indeed, each protein family is reported several times because in Supplementary Table S21, we report the occurrence of all sister-clade relationships for the Undinarchaeota, with a normalized sum of occurrences above 0.3. Since Undinarchaoeota can be split into sub-clades, there can be more than one sister relationship per tree. The raw output file, which also lists sister-relationships of all other archaeal clades, is part of our repository.

- The authors attempted to search hosts with a technique that I believe which might favour the detection of conserved, shared hosts across Udinarchaeota. Could this be that these species have developed specific interactions with different hosts along their diversification? In which case, could this explain why no HGT route is observable, unlike for other DPANN lineages? What is the author's view on this aspect?

Response: Our approach can only identify new host-symbiont systems if they have experienced high numbers of HGTs because: a) low numbers of exchange seem to occur between various microbial lineages sharing similar environments; b) these single gene trees have low signal-to-noise ratio such that for each individual tree, it is difficult to differentiate between HGT and lack of signal. Only if a signal is picked-up a certain number of times, would it be observable against the background noise.
However, we do see HGTs that only affect certain members of the Undinarchaeota but not others, because we count, for each lineage of interest, how many times it is split into distinct sub-lineages and what the closest sister-group of each of the sub-lineage is. For example, the tree in Figure 65, panel c) depicts a sister-relationship of the mevalonate kinase of one Undinarchaeota MAG with Chloroflexi, while the homologs of other Undinarchaeota emerge at a different position in the tree. (
in turn, we completely agree with the reviewer and based on our results have the hypothesis, that interaction partners differ at least on the level of marine versus aquifer Undinarchaeota. We now mention this possibility in the discussion more explicitly: "Altogether, in light of these results and since Naiad- and Undinarchaeales differ with respect to putative proteins involved in cell-cell interactions (Supplementary Tables S16-S19) and certain metabolic features (e.g. the presence versus absence of a complete lipid biosynthesis pathway) (Figure 3, Supplementary Material), it seems likely that members of these orders interact with different microbial partners." (**see lines 623-626)**

- Line 365-366, it is mentioned that Udinarchaeales could co-occur with some Dehalococcoidales. Could the authors find any signs of potential HGT between Chloroflexi and Udinarchaeota? How likely do the authors think it is that the potentially exchanged genes are not part of the core genes analysed?

Response: While we did indeed investigate the possibility of HGT between Chloroflexi and Undinarchaeota, we could not identify a significant fraction of genes exchanged between these groups. However, there are few examples such as the Mevalonate kinase (arCOG01028) (see above) in which certain members of the Undinarchaeota (i.e. one Undin-representative only) cluster with certain Chloroflexi. A tree of this protein is now shown in the new Figure S65. However, it remains to be determined whether this is indeed reflective of an interaction between members of these groups. We now discuss this in the main text in addition to the supplementary material. (**see lines 619-622 in main text as well as lines 646-658 in the supplementary information).**
Considering that we generated gene trees for all genes present in at least 3 Undinarchaeota genomes, and that gene sets are fairly consistent across members, we think it is unlikely that we missed a significant fraction of HGTs - yet low numbers of HGTs among certain Undinarchaeota and other microbial lineages cannot be excluded and could have been missed if not part of the core genes. However, these cases would not be numerous enough to be visible in the HGT plot. This is supported by our analysis of all archaeal protein families (not only those in at least three Undinarchaeota genomes), that revealed the same phylogenetic patterns of protein families for the various Undinarchaeota lineages and did not identify additional major routes of HGTs (see above).

Other comments

- Line 149: it is said that the "best-fitting models of sequence evolution" were used, yet it does not seem that this was statistically assessed e.g. using the "–m TEST" option of IQ-Tree. Maybe just say they are among the "most sophisticated models"?

Response: We thank the reviewer for pointing this out. We have now reformulated the sentence to "As compositionally heterogeneity across sites is a pervasive feature of archaeal sequence evolution[27], we used site-heterogeneous mixture models in our focal analyses in both maximum likelihood (IQ-TREE[50]) and Bayesian (PhyloBayes[51]) frameworks, in combination with alignment recoding and filtering of compositionally biased and fast-evolving sites." **(see line 263)**

- Line 151: I haven't seen any mention of statistical topology testing? Where did these analyses take place in the study? To test which topology against which?

Response: We apologize for this mistake. Due to the consistency of our results across our various analyses, we did not perform topology testing in the end but forgot to delete the mentioning of this in the main text. This has now been removed.

- Line 476: "We mined…" specify that the authors searched for Udinarchaeota?

Response: We have replaced "mined" by "searched for Undinarchaeota…"

- Line 494: To screen for contaminants, it is said that Diamond was used to assess taxonomic origin, but in Sup Mat, it is said line 34 that Blastp was used. So which is it?

Response: Thanks for noting this mistake. This was indeed a diamond search and we have corrected the respective mentioning in the Supplementary Material.

- Line 570: I don't understand what is meant by "combining TIGRFAM and GTDB"? What's the goal here? How is it ensured that TIGRFAM families meet the pre-requisites to build species trees (universality + unicity in genomes)? I believe some details are missing here.

Response: The GTDB marker set is composed of around 122 profiles selected from the PFAM and TIGRFAM databases. However, in our experience including a limited amount of profiles as queries can increase the risk of assigning distant paralogs. Therefore, we decided to create a larger set of profiles including all available TIGRFAM profiles to which we added missing PFAM profiles. Subsequently, we used all of these profiles in an

hmm-search against our proteomes of interest and extracted homologs for the proteins corresponding to the marker protein profiles. This was not worded clearly in the methods section and we therefore revised this section for better clarity. **(see lines 928-950)**

- In the Methods, I believe that the numerous supplementary tables should be referred to on more occasions, in order to help the reader find the datasets mentioned along the text. For instance, section starting on line 518: where is the list of genomes used as the "backbone dataset"? Section starting on line 559: refer to the tables S4 and S5? etc…

Response: We have now added the reference to the Supplementary Table (S23), that lists the backbone dataset and also added more references to Supplementary Tables at other occasions, where they were missing.

Figures
- Figure 1: The text in the box is inconsistent with the legend as it is said in the legend: "40% of the most heterog. sites were removed". So which is it?

Response: 10% of sites were removed and the figure was corrected accordingly.

- Figure 2: The legend could be improved by mentioning what's represented in grey. It seems it corresponds to what's missing from all genomes.

Response: Thanks for the suggestion, this was added to the legend.

- Figure 3 and Supp Figures 58-61: Indicating the Cluster 2 of DPANN on these figures could help (with brackets?) or instead, just mention in the figures legends that they are above Udinarchaeota within the DPANN rectangle?

Response: We now added some graphical aid in better distinguishing between Cluster 1 and Cluster 2 DPANN archaea.

- Figure 4: It is confusing, as there is a title to the figure in the legend "Potential HGTs..." and one on the figure: "Phylogenetic relationships of Undin. core proteins". I think the title in the legend is misleading. It's not only showing potential HGTs, but overall phylogenetic relatedness of the genes. Some could be interpreted as HGTs. I'd rather use the title displayed on the figure, that is more reflective of the figure content.

Response: We agree with this suggestion and changed the caption accordingly.

- Table 1. In the table legend, "parentheses" should replace "brackets".

Typos
line 71: "artefacts", line 82: "artifacts" => pick one?
line 71: "branch"
line 132: "marker"
line 388: "affected"
line 695 "sequences" missing in "with <1000"

Response: We have corrected these typos, thank you very much for pointing them out.

Reviewer #2 (Remarks to the Author):

This manuscript makes an impressive contribution to our understanding of DPANN evolution and ecology and is clearly the product of extensive and well performed analysis, even if most of it ended up in the huge supplementary information section. Furthermore, the analysis of HGT and how it affects the phylogeny of currently disputed archaeal groups is highly valuable for the field.

I am not sure why in the age of online only publications manuscripts must be kept so short, and there are sections in the supplementary information, especially those describing gene content, that should have been in the main text, IMHO.

Response: thank you very much for this kind and supportive assessment.

I have only minor points, see below.

"Nanoarchaeota are ectosymbionts of TACK archaea" - it would be better here to mention the specific lineages within TACK or at least spell out TACK for the reader.

Response: We have replaced TACK archaea by "various Crenarchaeota such as for example *Ignicoccus hospitalis*, Sulfolobales Acd1 and Acidilobus sp. 7A".

Table 1 - strain heterogeneity is not clearly presented

Response: The caption was changed to better describe the table.

"low ranking makers were highly incongruent" - should be "... markers..."

Response: Thank you for pointing this out. We have corrected this now.

"clan" and "clanhood" are relatively recent terms in phylogenetics that most readers will be unfamiliar with and should be introduced.

Response: This has been introduced better now.

Since the archaeal root cannot be reliably inferred are there synapomorphies supporting DPANN as a clan?

Response: We have tried to identify potential synapomorphies shared across all DPANN lineages but did not find anything that would support their monophyly. I.e those protein families present in a large variety of DPANN (>80%) are also present in other archaeal genomes. To illustrate this, we have now added two additional columns in Table S9, i.e. BQ and BR. It is possible that the absence of clear indications for synapomorphies is due to the large variation of genome completeness and gene sets between cluster 1 and 2 DPANN.

"Nanohaloarchaeota and Halobacteria shared a large amount of analyzed 345 proteins horizontally (12.4% and 7%)" - unclear what the two different % refer to in this sentence.

Response: We apologize that this was not clear enough. It means that in 12.4% and 7% of all sister-hood relationships with a normalized occurrence of >0.3, (certain) Nanohaloarchaeota clades branched with Halobacteria and certain Halobacteria branched with Nanohaloarchaeota, respectively. For clarity, we now rephrased this to: Furthermore, our phylogenies suggest that in several instances Nanohaloarchaeota and Halobacteria form sister-groups suggesting that these lineages have exchanged proteins horizontally (i.e. 12.4% and 7% of all sister-hood relationships for these two clades, respectively) (Figure 4), which is in agreement with our marker gene analyses (Supplementary Tables 4-5). This has now been worded more clearly. **(see lines 579-584)**

Either figure 4 is not adequately explained or there are lineages for which there are more proteins that originate from HGT than by shared ancestry (for example the Halobacteria). Such cases have been shown in bacteria (for example the Thermotogales), but not in archaea, so if this is indeed the case for multiple lineages the text should say something about it.

Response: We apologise; Figure 4 was not adequately explained: for each archaeal clade, it shows the relative frequencies with which each other archaeal clade was recovered as its sister group, averaged over all genes and bootstrap replicates. One subtlety of the approach is that an archaeal clade cannot be sister to itself, if these two sister lineages are both monophyletic, because they would be considered as a single clade in the analysis.

If the sister lineage is itself taxonomically mixed, the relatively frequencies of each of the component taxa will be increased proportionally. Thus, the null expectation for a clade that experiences no gene transfer or gene tree error would be that it should clade with the closest sister lineage in the archaeal species tree. For example, if we have one monophyletic clade comprised of the Haloarchaeota, we would report its closest sister-lineage, which in most cases would be a euryararchaeal lineage as expected. If Haloarchaeota are split into two or more lineages in a given tree, we would report every supported sister-lineage. In the end, we plot for each lineage the fraction of how many times a certain sister-hood relationship was observed relative to the total amount of observations. We have now clarified this in the methods, figure legend and main text.
We have now more clearly explained Figure 4 in the methods, results and figure legend. (**see lines 579-584, 748-757, 1100-1124).**

REVIEWERS' COMMENTS:

Reviewer #1 (Remarks to the Author):

All my concerns/suggestions/questions were addressed in this revised version of the paper, and I want to thank the authors for their detailed replies, and work on the manuscript. I now fully recommend this article for publication.

Here are a few minor typos/suggestions:

Line 55: maybe remove extra “and”?
L. 61: italicize species names.
L. 88: include a missing “as”? (“referred to as the”)
L. 327: one occurrence left for “artifact” instead of “artefact”. Should “artifactual” on line 600 also be changed for “artefactual”?
L. 354: remove an extra “and”?
L. 558: I believe this sentence could start without the “But”.
L. 608: replace the unusual word “clading” by a more classical “grouping”?
L. 824: “used genomes” seems odd to me? Please check. Maybe “genomes used” instead? Same comment on line 828.
L. 860-862: HMM is an acronym and should thus be spelled in upper case: “HMM”.
Line: 864-865: this hmmsearch command-line seems odd to me (“Table”?). The authors may want to check it.

Reviewer comments

Line 55: maybe remove extra “and”?

Response: We have changed this as suggested

L. 61: italicize species names.

Response: We have changed this as suggested

L. 88: include a missing “as”? (“referred to as the”)

Response: We have changed this as suggested

L. 327: one occurrence left for “artifact” instead of “artefact”. Should “artifactual” on line 600 also be changed for “artefactual”?

Response: We have changed this as suggested

L. 354: remove an extra “and”?

Response: We have changed this as suggested

L. 558: I believe this sentence could start without the “But”.

Response: We have changed this as suggested (now line 405)

L. 608: replace the unusual word “clading” by a more classical “grouping”?

Response: We have changed this as suggested (now line 438)

L. 824: “used genomes” seems odd to me? Please check. Maybe “genomes used” instead? Same comment on line 828.

Response: We have changed this as suggested

L. 860-862: HMM is an acronym and should thus be spelled in upper case: “HMM”.

Response: We have changed this as suggested

Line: 864-865: this hmmsearch command-line seems odd to me (“Table”?). The authors may want to check it.

Response: We have checked this and verified that it is accurate. It looks slightly different than usual because this command calls a small script (rather than directly calling hmmsearch) that is based on the hmmsearch command but includes some additional parsing steps to make the output more readable. The sentence was modified slightly to clarify this and the script is provided in the repository as well.
